# Kilogram scale facile synthesis and systematic characterization of a Gd-macrochelate as T_1_-weighted magnetic resonance imaging contrast agent

**DOI:** 10.1186/s12951-024-02394-8

**Published:** 2024-04-09

**Authors:** Meng Shi, Wei Xiong, Jie Feng, Lihe Wu, Jing Yang, Yudie Lu, Xuanyi Lu, Qingdeng Fan, Hemin Nie, Yunlu Dai, Chenggong Yan, Ye Tian, Zheyu Shen

**Affiliations:** 1 School of Biomedical Engineering, Southern Medical University, 1023 Shatai South Road, Baiyun, Guangzhou, 510515 Guangdong China; 2 Medical Imaging Center, Nanfang Hospital, Southern Medical University, 1023 Shatai South Road, Baiyun, Guangzhou, 510515 Guangdong China; 3 Department of Biomedical Sciences, College of Biology, Hunan University, 52 Tianmu Road, Yuelu, Changsha, 410082 Hunan China; 4 Faculty of Health Sciences and MoE Frontiers Science Center for Precision Oncology, University of Macau, Macau SAR, 999078 China

**Keywords:** Contrast agents (CAs), Kilogram scale facile synthesis, Remarkable relaxivity, Magnetic resonance imaging (MRI), Excellent biocompatibility

## Abstract

To overcome the problems of commercial magnetic resonance imaging (MRI) contrast agents (CAs) (*i.e.*, small molecule Gd chelates), we have proposed a new concept of Gd macrochelates based on the coordination of Gd^3+^ and macromolecules, *e.g.*, poly(acrylic acid) (PAA). To further decrease the *r*_2_/*r*_1_ ratio of the reported Gd macrochelates that is an important factor for *T*_1_ imaging, in this study, a superior macromolecule hydrolyzed polymaleic anhydride (HPMA) was found to coordinate Gd^3+^. The synthesis conditions were optimized and the generated Gd-HPMA macrochelate was systematically characterized. The obtained Gd-HPMA29 synthesized in a 100 L of reactor has a *r*_1_ value of 16.35 mM^−1^ s^−1^ and *r*_2_/*r*_1_ ratio of 2.05 at 7.0 T, a high Gd yield of 92.7% and a high product weight (1074 g), which demonstrates the feasibility of kilogram scale facile synthesis. After optimization of excipients and sterilization at a high temperature, the obtained Gd-HPMA30 formulation has a pH value of 7.97, osmolality of 691 mOsmol/kg water, density of 1.145 g/mL, and viscosity of 2.2 cP at 20 ℃ or 1.8 cP at 37 ℃, which meet all specifications and physicochemical criteria for clinical injections indicating the immense potential for clinical applications.

**Graphical Abstract:**

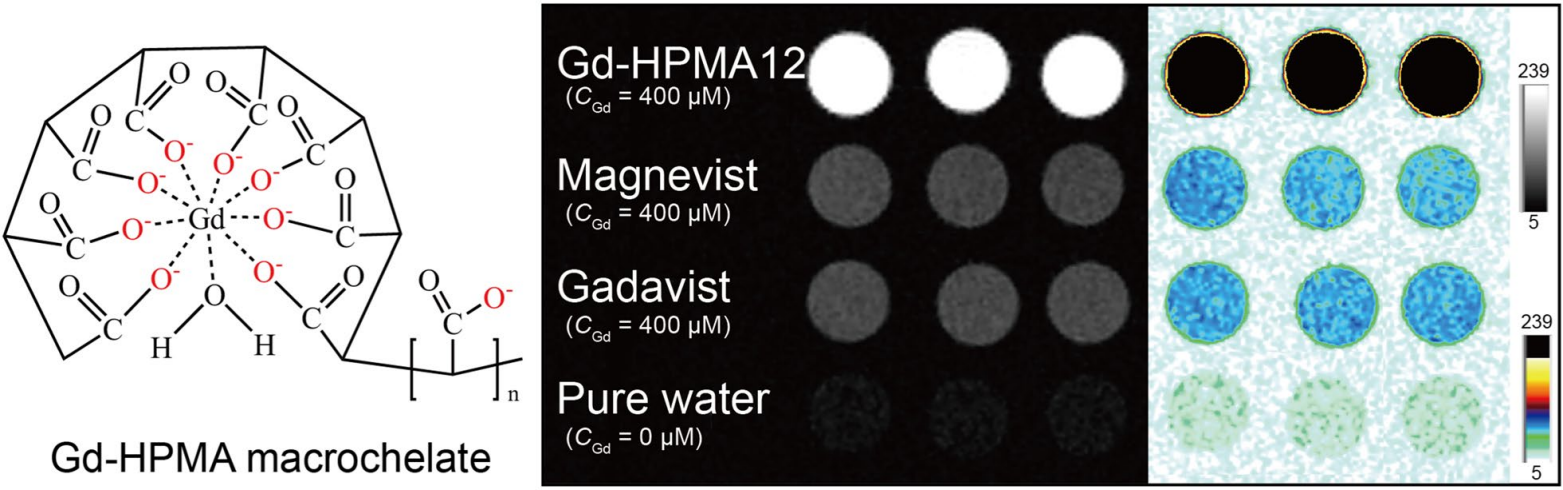

**Supplementary Information:**

The online version contains supplementary material available at 10.1186/s12951-024-02394-8.

## Introduction

Magnetic resonance imaging (MRI) has become a well-known clinical diagnosis apparatus with the advantages of safety, non-invasiveness, multi-parameters, and high-quality three-dimensional images of soft tissue [1–3]. Millions of MRI exams are performed annually all throughout the world and 40–50% of them have used contrast agents (CAs) to improve diagnostic accuracy [4–6]. According to their contrast generating mechanisms, MRI CAs are mainly classified into two categories: *T*_1_-weighted CAs (*i.e*., paramagnetic and positive contrast agents) and *T*_2_-weighted CAs (*i.e*., super-paramagnetic and negative contrast agents). *T*_2_-weighted CAs predominantly shorten protons’ transverse (*T*_2_) relaxation times and provide darkened MRI images, which are so adverse to identifying tissues or lesions at certain pathogenic conditions that most productions of *T*_2_-weighted CAs have ceased on current market [7–9]. On the contrary, gadolinium(III)-based contrast agents (GBCAs) have been the most successful *T*_1_-weighted CAs so far and there is no doubt they dominate the market for MRI CAs. Gd^3+^ has become the most widely used metal center to form *T*_1_-weighted CAs with brighter MRI images by shortening protons’ longitudinal (*T*_1_) relaxation times and increasing the intensity in regions where they distribute [10, 11]. Eight of the twelve GBCAs that FDA has approved are still readily accessible in American pharmacies, including Magnevist^®^ (Gd-DTPA), Gadavist^®^ (Gd-DO3A-butrol), Dotarem^®^ (Gd-DOTA), and ProHance^®^ (Gd-HP-DO3A). For years the use of these GBCAs was considered very safe until a strong link between GBCAs and nephrogenic systemic fibrosis (NSF) was identified in 2006 [12–14]. Furthermore, increasing evidence since 2013 suggests that Gd^3+^ is permanently deposited in the central nervous system (CNS) after patients receiving GBCAs [15–17]. NSF and deposited in CNS are strongly attributable to gadolinium exposure: although the precise mechanisms of NSF onset remain undetermined, it seems that a complex of Gd^3+^ and the metalloprotein ferritin may contribute to the Gd deposition in the CNS [18, 19].

As a result, the growing concerns over the safety of MRI CAs are driving researchers to develop much safer products. One typically adopted strategy is to develop and design CAs with high longitudinal relaxivity (*r*_1_) values and low *r*_2_/*r*_1_ ratios (*r*_2_ is transverse relaxivity), so they can be administered at a much lower Gd dosage than GBCAs. According to Solomon-Bloembergen-Morgan paramagnetic relaxation theory [20, 21], we can manipulate the paramagnetic relaxation enhancement (PRE) effect of Gd chelates to alter the observed MR signals.

1 $$r_{1} = r_{1}^{OS} + r_{1}^{IS}$$

2 $$r_{1}^{IS} = \frac{{q/\left[ {H_{2} O} \right]}}{{T_{1m} + \tau_{m} }}$$

3 $$\frac{1}{{T_{1m} }} = \frac{{C_{1} }}{{r_{GdH}^{6} }} \times \frac{{\tau_{c}^{ - 1} }}{{\tau_{c}^{ - 2} + 4\pi^{2} \omega_{H}^{2} }}$$

4 $$\tau_{c}^{ - 1} = \tau_{r}^{ - 1} + \tau_{m}^{ - 1} + T_{1e}^{ - 1}$$

The *r*_1_ can be factored into outer-sphere relaxivity $r_{1}^{OS}$ and inner-sphere relaxivity $r_{1}^{IS}$ (Eq. 1). Despite arising from PRE on water molecules that are hydrogen bound to the Gd chelate and that are further away, $r_{1}^{OS}$ is still not fully understood [22, 23]. The inner-sphere relaxivity ($r_{1}^{IS}$) is related to three key terms: hydration number (*q*), water residency time ($\tau_{m}$), and *T*_1_ of Gd(III)-bound water (*T*_1m_), as shown in Eq. 2. *T*_1m_ is described by Eq. 3 where *C*_1_ is a constant, $r_{GdH}$ is the Gd(III)-water proton distance, $\tau_{c}$ is the correlation time for magnetic fluctuation induced by Gd(III), and $\omega_{H}$ is the Lamor frequency of proton. Because of a 100-fold smaller rotational correlation time ($\tau_{r}$, about 0.1 ns at 0.5 T) compared with $\tau_{m}$ or $T_{1e}$ (electronic relaxation time), $\tau_{c}$ is dominated by $\tau_{r}$ (Eq. 4) [24]. From the above discussion, it can be extrapolated that increasing $\tau_{r}$ can significantly enhance *r*_1_. Specifically, high-molecular-weight and slowly tumbling molecules with large $\tau_{r}$ achieve higher *r*_1_ than GBCAs with low molecular weight (< 1000), whose *r*_1_ values are only 3–8 mM^−1^ s^−1^. Additionally, larger complexes tend to be cleared more slowly in the body compared to small molecules, which allows enhanced accumulation and retention, further enhancing the observed MR signal. However, a slow clearance time increases the risk of Gd exposure.

In this regard, we have made efforts to develop promising CAs with improved MRI performances over the years, and have proposed a new concept of Gd macrochelates based on the coordination of Gd^3+^ and macromolecules [25]. Typically, the macromolecule poly (acrylic acid) (PAA, Mw = 5100 Da) reacts with Gd^3+^ to form the Gd-PAA macrochelate that tumbles slower in solution, hence increasing $\tau_{r}$ and dramatically enhancing *r*_1_. The obtained Gd-PAA macrochelate exhibits a high *r*_1_ value (56.23 ± 1.69 mM^−1^ s^−1^, 3.0 T; 17.18 mM^−1^ s^−1^, 7.0 T) and a low *r*_2_/*r*_1_ ratio (1.54 ± 0.03, 3.0 T; 5.39, 7.0 T), which have corroborated the above-mentioned inference. Although the Gd-PAA macrochelate synthesized in 20 L of reactor still possess outstanding relaxation properties (*r*_1_ = 50–54 mM^−1^ s^−1^, *r*_2_/*r*_1_ = 1.4–1.6, 3.0 T; *r*_1_ = 16–17 mM^−1^ s^−1^, *r*_2_/*r*_1_ = 4.2–5.5, 7.0 T), there remain the following issues to be resolved: (1) the *r*_2_/*r*_1_ ratios are not amply low, which is an important factor for *T*_1_ imaging; (2) 20 L of reactor is too small for industrial manufacture; (3) the excipients of the Gd-PAA formulation are not investigated to control the physicochemical properties (*i.e.*, pH value, osmolality, viscosity, and density) and then meet the requirements for clinical injection.

**Scheme 1:**
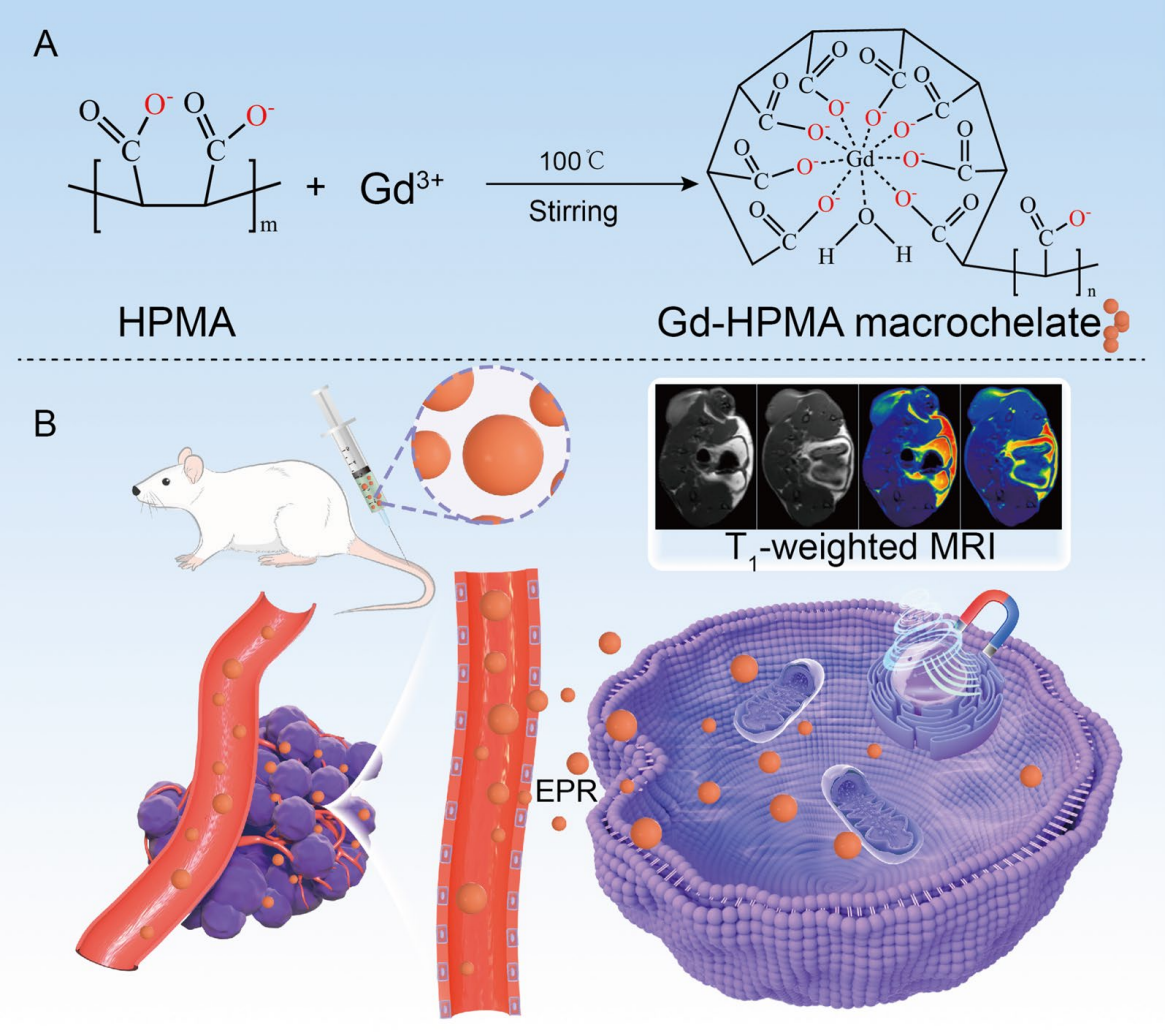
**A** Schematic illustration for kilogram scale facile synthesis of Gd-HPMA macrochelates. **B** The Gd-HPMA macrochelate accumulates at the tumor through the EPR effect, which can be used for high contrast *T*_1_-weighted MRI

To overcome these problems, after screening a library of macromolecules for Gd^3+^ coordination, we found a superior macromolecule, *i.e.*, hydrolyzed polymaleic anhydride (HPMA), generating Gd-HPMA macrochelate. The Gd-HPMA macrochelate should possess high *r*_1_ value because of: (i) the presence of hydration of the metal center (q = 1 or > 1); (ii) the high P_m_ (*i.e.*, the high mole fraction of water coordinated to the metal center) resulted from the super-hydrophilicity of HPMA; (iii) the high τ_R_ (*i.e.*, slow tumbling of the large molecule in aqueous solution); (iv) the reasonable value of the residence time (*i.e.*, τ_M_) [25]; (v) the presence of a strong outter sphere contribution. In addition, as one of the synthesis conditions, the molar ratio of Gd^3+^ to HPMA is smaller than 1.0. Therefore, there is only one Gd^3+^ in the structure of Gd-HPMA macrochelate as shown in Scheme 1A.

HPMA with one more carboxyl group in the polymer repeating unit compared with PAA enhances the electrostatic repulsion between the Gd-HPMA macrochelates and thus improves the dispersity of the Gd-HPMA macrochelates, which enables the lower saturation magnetization (Ms) and *r*_2_ value (Eq. 5).

5 $$\frac{1}{{T_{2} }}{ = }\frac{{{\text{C}}_{2} \gamma^{2} V^{ * } r^{2} }}{{D\left( {1 + L/r} \right)}} \times M_{s}^{2}$$

As a result, the Gd-HPMA macrochelate achieves a very high *r*_1_ value (50.46 ± 1.01 mM^−1^ s^−1^, 3.0 T; 16.36 ± 0.87 mM^−1^ s^−1^, 7.0 T) and an ultralow *r*_2_/*r*_1_ ratio (1.21 ± 0.06, 3.0 T; 2.17 ± 0.12, 7.0 T), which are both very good for *T*_1_ imaging (Scheme 1B). The Gd-HPMA macrochelate synthesized in a 100 L of reactor exhibits excellent relaxation properties (*r*_1_ = 16.35 mM^−1^ s^−1^, *r*_2_/*r*_1_ = 2.05, 7.0 T) and a high product weight (1074 g), while Gd-PAA synthesized in a 20 L reactor weighs only 98 g. After investigation of the excipients, the obtained Gd-HPMA formulation has a pH value of 7.97, osmolality of 691 mOsmol/kg water, density of 1.145 g/mL and viscosity of 2.2 cP at 20 ℃ or 1.8 cP at 37 ℃, which meet all specifications and physicochemical criteria for clinical injections [26–28]. More importantly, the excipients and sterilization at a high temperature have no adverse effect on the relaxation properties of Gd-HPMA30 (*r*_1_ = 16.30 mM^−1^ s^−1^, *r*_2_/*r*_1_ = 2.07, 7.0 T).

## Methods

### Synthesis of Gd-HPMA

HPMA (Mw = 1200 Da) solution (4.0 mg/mL, 20 mL) was purged using N_2_ to remove O_2_ for 60 min. The polymer solution was kept at room temperature (25 ℃), or heated to reflux (100 ℃). After that, GdCl_3_ (62.5–500 mM, 0.40 mL) solution was injected into the mixture. The reaction lasted for 60 min under magnetic stirring at room temperature or 100 ℃ to obtain Gd-HPMA macrochelates. Finally, the solutions were cooled down to room temperature. The obtained Gd-HPMA macrochelates were purified by membrane dialysis (Mw cut-off 3.5 kDa) against Milli-Q water for three days with water changes twice a day. The purified Gd-HPMAs were concentrated via rotary evaporation.

Large-scale facile synthesis of the Gd-HPMA macrochelates was conducted in a 2.0, 20, or 100 L of reactor via the above-mentioned procedures with optimized conditions.

### Adjuvant investigation

The obtained Gd-HPMA macrochelate after dialysis had a pH value of 7.89 (*C*_Gd_ = 1.25 mM). HCl (0.02 M) and Tris (0.1%) were mixed at 28:100 (v/v), and the mixture solution HCl-Tris had a pH of 7.21. Then, Tris–HCl and Gd-HPMA (*C*_Gd_ = 1.25 mM) were mixed at 20:80 (v/v). The mixture is then concentrated by rotary evaporator to obtain Gd-HPMA with high Gd concentration. The Gd-HPMA with high Gd concentration was diluted to obtained Gd-HPMA30 (*C*_Gd_ = 100 mM) for physicochemical properties investigation. The Gd concentration was measured by ICP-OES (Thermo Fisher Scientific, iCAP PRO Series), pH value was measured by pH meter (lei-ci, PHS-2F), osmolality was measured by automatic cryoscopic osmometer (neuronbc, FPOSM-V), viscosity was measured by rotational viscometer (shjingmi, NDJ-5S), and density was measured by liquid densimeter (shjingmi, CA-120YT).

### Synthesis of R6G-loaded Gd-HPMA12

4.0 mL of Gd-HPMA12 (*C*_Gd_ = 2.0 mM) was mixed with 0.70 mL of R6G (1.0 μM) under magnetic stirring at 25 ℃ (R6G is adsorbed onto Gd-HPMA through hydrogen bonding). After 24 h, the obtained R6G-Gd-HPMA12 was purified by membrane dialysis (Mw cut-off 3.5 kDa) against Milli-Q water for 3 days with water changes twice a day to remove the unloaded R6G. The finally obtained R6G-Gd-HPMA12 was kept in a fridge for next use.

### General measurement

Gd concentrations (*C*_Gd_) of the solutions were measured using an inductively coupled plasma optical emission spectrometer (ICP-OES, iCAP PRO Series, Thermo Fisher Scientific). The structure of the Gd-HPMA was verified by Fourier transform infrared spectrometer (FT-IR, Nicolet IS50, Thermo Fisher Scientific). Field-dependent magnetization curve of the Gd-HPMA12 was measured by physical property measurement system (PPMS) at 300 K.

### Release behaviors of Gd from Gd-HPMAs

The Gd-HPMA12 (*C*_Gd_ = 1.0 mM, 10 mL) was transferred into a dialysis bag, which was then placed in 500 mL DMEM with 10% FBS, or saline solution of pH 2.0, pH 7.0, or pH 7.0 with double amount of Zn^2+^ compared with Gd in Gd-HPMA12. The pH of saline solution is adjusted using HCl. The saline solutions were stored at 37 ℃. At predetermined time intervals, 10 mL of the saline solutions were taken and digested for ICP-MS analysis. The Gd release behavior at different conditions was monitored via a plot of the cumulative released Gd content (*i.e.*, the molar percentage of the released Gd to the total amount of Gd in Gd-HPMA as a function of incubation time. The release of Gd from Magnevist^®^ or Gadavist^®^ (*C*_Gd_ = 1.0 mM, 10 mL) in 500 mL PBS was measured using the same method.

### Cellular uptake

Cellular uptake of nanoparticles was measured via laser scanning confocal microscopy (LSCM), flow cytometry, ICP, and MRI.

By LSCM: 4T1 cells were cultured in confocal dishes at 1.0 × 10^4^ cells/mL of density for 24 h. After that, the growth media were replaced with 0.50 mL of fresh one without or with R6G-Gd-HPMA12 (*C*_Gd_ = 400 μM). The cells were incubated for 4.0 h, washed using PBS twice, fixed using 4.0% paraformaldehyde for 30 min, permeabilized using 0.10% Triton X-100 for 5.0 min, blocked using 1.0% BSA for 30 min, and finally stained with a mixture of 4ʹ,6-diamidino-2-phenylindole (DAPI, Beyotime Biotechnology) and Phalloidin-FITC solution for 30 min. The obtained cells were observed by a LSCM (Nikon ECLIPSE Ti2).

By flow cytometry: 4T1 cells were seeded in a 6-well plate (2.0 × 10^5^ cells/well), cultured overnight, and incubated with R6G-Gd-HPMA12 for 4.0 h (*C*_Gd_ = 400 μM). The cells were then washed with cold PBS twice, trypsinized and harvested by centrifugation at 800 ×*g* for 3.0 min. The cells were resuspended in PBS (200 μL), and then detected by flow cytometry (BD FACSAria III, USA).

By ICP: 4T1 cells in complete DMEM medium were seeded in 6-well plates at 2.0 × 10^5^ cells/well of density, and incubated at 37 °C for 24 h. The growth medium was then replaced with a fresh one without or with Gd-HPMA12 (*C*_*Gd*_ = 400 μM). After incubation at different times (0.25, 0.50, 1.0, or 2.0 h), the cells were washed twice using PBS, treated using trypsin for 3.0 min, and then centrifuged at 800 × g for 3.0 min. The finally obtained cells were digested for Gd detection utlizing ICP-MS.

By MRI: 4T1 cells were seeded in 6-well plates at 2.0 × 10^5^ cells/well of density, cultured for 24 h, and then treated with Gd-HPMA12 (*C*_Gd_ = 400 μM) for different times (0.25, 0.5, 1.0, or 2.0 h) at 37 °C. Next, the cells were harvested in 0.5 mL centrifuge tubes, and 0.20 mL of agarose solution (0.80 wt%) was added to fix the cells. *T*_1_-weighted MR images were acquired using a 3.0 T MRI scanner under following parameters: TE = 8.2 ms, TR = 500 ms.

### MTT assay

Cell viabilities were measured utilizing the MTT assay. Typically, 150 μL of 4T1 cells in the complete DMEM medium were seeded into each well of 96-well plates at a concentration of 1.0 × 10^4^ cells/mL, and allowed to adhere overnight. The DMEM medium was then replaced with a fresh DMEM medium without FBS containing various concentrations (*C*_*Gd*_ = 3.90, 7.80, 15.6, 31.3, 62.5, 125, 250 µg/mL) of Gd-HPMA12. After 24 h of incubation at 37 °C, the growth medium was replaced with a complete medium, and 10 μL of MTT (5.0 mg/mL in PBS) were added to each well of the 96-well plates. After further 4.0 h of incubation, the growth medium was removed and the generated formazan crystals were dissolved using 100 μL of DMSO. The absorbance was obtained at 490 nm of wavelength on a multi-mode microplate reader (Synergy H1, BioTek Instruments, USA).

### Pharmacokinetics and biodistribution of Gd-HPMA12 in vivo

For pharmacokinetic studies, healthy female Balb/c mice (20 ± 2 g) were injected (*i.v.*) with Gd-HPMA12 via tail vein (Gd dosage = 5.0 mg/kg, *n* = 3). The blood samples (around 20 μL) were drawn from retro-orbital sinus at predetermined times (2.5, 5.0, 10, 15, 25, 40, 60, 80, 110, 150, 210 min) after injection, and then thoroughly digested using concentrated nitric acid. The liquid was evaporated under heating. The sample residues were dissolved in 2.0% diluted nitric acid, and the concentration of Gd (*C*_Gd_) was detected by ICP-OES.

For biodistribution analysis, the 4T1 tumor-bearing mice were injected (*i.v.*) with Gd-HPMA12 (Gd dosage = 5.0 mg/kg, n = 3). The main organs (heart, liver, spleen, lung, kidney, muscle, bone, brain) and tumors were excised at 1.0 h or 12 h post-injection, and then completely digested in heated concentrated nitric acid. The liquid was evaporated under heating. The sample residues were dissolved in 2.0% diluted nitric acid, and the *C*_Gd_ was measured by ICP-OES. The biodistribution of Gd was calculated as a percentage of injected dose per gram of tissue (I.D.%/g).

### MRI examination for Gd-HPMA macrochelates in vivo

In vivo* T*_1_-MRI evaluation of Gd-HPMA12 was performed using a 3.0 T clinical MRI scanner with an 8-channel mouse coil. The parameters were shown as: TR/TE = 500/8.4 ms, FOV = 50 × 50 mm^2^, matrix size = 252 × 248, number of averages = 4, scan duration = 232 s. The contrast agent Gd-HPMA12 (dosage = 5.0 mg Gd/kg body weight, *i.e.*, 43.2 mg Gd-HPMA/kg body weight) was injected into 4T1-bearing mice via tail vein. Three representative slices were selected close to the central region of the tumor. The region of interest (ROI) encompassed the entire tumor area on each slice, and the mean ± SD was calculated from the three slices.

### Hemolysis analysis of Gd-HPMA macrochelates

For hemolysis assay, red blood cells were first isolated by centrifugation of fresh blood from Balb/c mice (250 ×*g*, 10 min). The concentration of the collected blood cells was then diluted to 2.0% (v/v). The *C*_Gd_ of Gd-HPMA12 was respectively adjusted to 0–500 μg/mL using PBS. After that, 500 μL of the Gd-HPMA12 solutions with various *C*_Gd_ were respectively added into 500 μL of blood cells, and the mixtures were immediately incubated in 37 °C of water bath for 4.0 h. Under the same conditions, red blood cells were respectively mixed with pure water or PBS (pH = 7.4) as a positive or negative control. The samples were taken out and centrifuged (250 ×*g*, 15 min) to remove intact red blood cells. 100 μL of the supernatants were added into a 96-well plate, and the absorbance was measured at 545 nm using a microplate reader (Synergy H1, BioTek Instruments). Finally, the percentage of hemolysis was determined as (A_sample_ − A_PBS_)/(A_pure water_–A_PBS_) * 100%, where A_sample_, A_pure water_, and A_PBS_ are the absorbance of the samples, the completely lysed red blood cells in pure water, and zero hemolysis in PBS. Three parallel groups were tested for each concentration.

### Excretion study of Gd-HPMA macrochelates

The SD mice were injected (*i.v.*) with Gd-HPMA12 (Gd dosage = 5.0 mg/kg). The excreta (*i.e.*, urine and feces) were collected every 8.0 h within 24 h after injection, and then completely digested in heated concentrated nitric acid. The liquid was evaporated under heating. The sample residues were dissolved in 2.0% diluted nitric acid, and the *C*_Gd_ was measured by ICP-OES. The Gd content was calculated as a percentage of injected dose of urine or feces (I.D.%) for excretion analysis.

### Biosafety evaluation of Gd-HPMA macrochelates

The biosafety of Gd-HPMA12 was investigated on healthy Balb/c mice by blood routine analyses and histological analyses of main organs.

Blood routine analyses: Balb/c mice were randomly divided into control group and experimental group. Experimental mice were intravenously injected with Gd-HPMA12 (Gd dosage = 10.0 mg/kg, 200 µL). Blood samples were harvested for blood routine analyses at day 1.0, 7.0 or 21. The mice injected with PBS were used as blank controls.

Histological analyses of main organs: the major organs of the mice were excised and subjected to histological analyses at day 2.0 or 30 post-injection (*i.v.*) of PBS or Gd-HPMA12 (Gd dosage = 10.0 mg/kg, 200 µL). Hematoxylin and eosin (H and E) staining was performed on major organs including the heart, liver, spleen, lung and kidney, which were observed by a microscope (Nikon ECLIPSE Ci-L plus).

### Acute systemic toxicity of Gd-HPMA macrochelates

A group of laboratory SD mice (25 adult males and 25 adult females; 6 weeks old; outbred strain) were obtained from Laboratory Animals of Southern Medical University. They were acclimatized in individual cages for 7–10 days. Then they were divided into 5 groups randomly, with 10 animals in each group, including 5 males and 5 females. Animals in each group were intravenously injected with Gd-HPMA30 at a dosage of 150, 75.0, 30.0, 12.0, or 5.00 mg/kg. The mice were observed every 30 min for 8.0 h post-injection to see if they died. The LD_50_ was calculated using Bliss Analysis. Median effective dose (ED_50_) quals injection or standard dose.The TI was calculated according to the following equation: TI = LD_50_/ED_50_.

## Results and discussion

### Synthesis optimization and MRI performance of Gd-HPMA macrochelates

Gd-HPMA macrochelates with remarkable relaxivities were synthesized by the carboxyl coordination reaction between Gd^3+^ and HPMA (Mw = 1200 Da). The linear regression analyses, depicting the dependencies of *T*_1_ relaxation rate and *T*_2_ relaxation rate on *C*_Gd_ for Gd-HPMA1-9 macrochelates at 3.0 T, are presented in Additional file 1: Figures S1, S2, respectively. The synthesis conditions and corresponding *r*_1_ and *r*_2_ values (*i.e.*, the slopes) are shown in Table S1. The Gd/HPMA molar ratio was meticulously optimized to 0.75 due to the relatively high *r*_1_ value (48.14 ± 0.56 mM^−1^ s^−1^) and low *r*_2_/*r*_1_ ratio (1.19 ± 0.04) at 3.0 T (Additional file 1: Figure S3A). The chemical stability of the Gd-HPMA macrochelates was evaluated by measuring the *r*_1_ and *r*_2_/*r*_1_ as a function of pH ranging from 5.0 to 10.0 at 3.0 T (Figure S3B). The comparable *r*_1_ values demonstrate the outstanding chemical stability of Gd-HPMAs over the entire range of investigated pH. The optimal pH value for synthesis is determined to be 9.0 because a more strongly alkaline condition results in the ionic interaction between the Gd^3+^ and carboxyl groups [25]. In addition, the *T*_1_-weighted MR images of Gd-HPMA1-9 at 3.0 T are shown in Additional file 1: Figure S4, indicating the remarkable MRI properties.

Furthermore, a comprehensive exploration was conducted regarding the influence of reaction temperature and nitrogen atmosphere on the relaxivities. Additional file 1: Figures S5, S6 show the linear fitting of the *T*_1_ relaxation rate or *T*_2_ relaxation rate of Gd-HPMA10-13 macrochelates with *C*_Gd_ at 3.0 T and 7.0 T, respectively. The synthesis conditions and corresponding *r*_1_ and *r*_2_ values (*i.e.*, the slopes) are shown in Additional file 1: Table S2. The comparable relaxivities of Gd-HPMA10-13 macrochelates suggest that alterations in reaction temperature and the presence or absence of a nitrogen atmosphere have failed to exert any discernible influence upon the Gd^3+^ chelation process with HPMA. Considering non-nitrogen atmosphere and room temperature conditions are conducive to subsequent large-scale synthesis, the Gd-HPMA12 macrochelate (*r*_1_ = 50.46 ± 1.01 mM^−1^ s^−1^, *r*_2_/*r*_1_ = 1.21 ± 0.06, 3.0 T; *r*_1_ = 16.36 ± 0.87 mM^−1^ s^−1^, *r*_2_/*r*_1_ = 2.17 ± 0.12, 7.0 T) was considered as the optimal sample for the following characterizations. Significantly, the ascertained *r*_1_ value of the Gd-HPMA12 macrochelate stands notably superior, eclipsing commercial GBCAs by an order of magnitude [29, 30]. That’s because the high molecular weight of Gd-HPMA12 can limit the Gd tumbling, increase rotational correlation times and induce strong *T*_1_ water proton spin relaxation, thus exhibiting an excellent *r*_1_ value compared with commercial *T*_1_-weighted MRI CAs [30, 31].

**Fig. 1:**
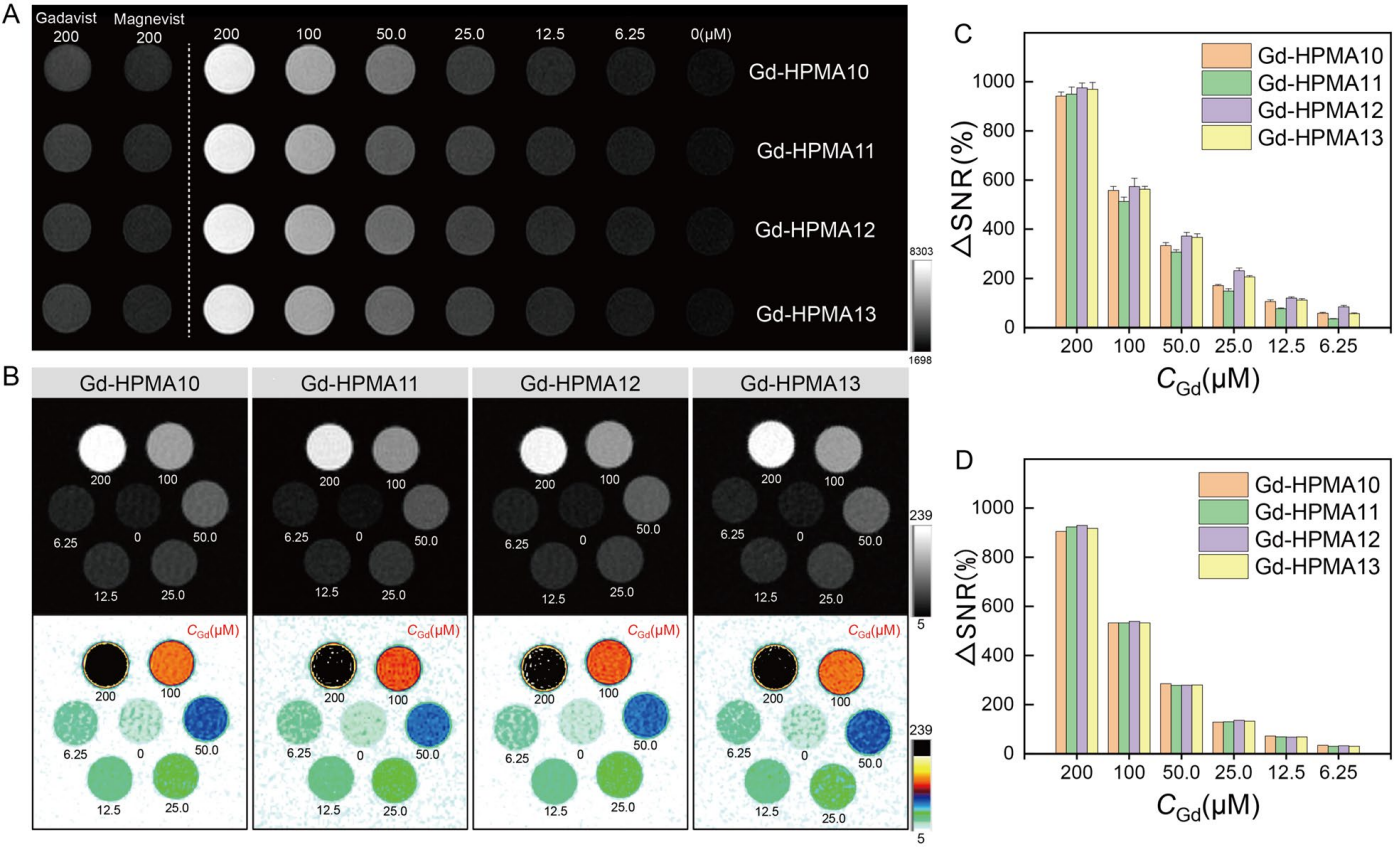
*T*_1_-weighted MR imaging of Gd-HPMA macrochelates at various concentrations. **A**: *T*_1_-weighted MR images of Gd-HPMA macrochelates with various *C*_Gd_ (0 ~ 200 μM) compared with the commercial Gadavist^®^ and Magnevist^®^ (200 μM) observed by a 3.0 T clinical MRI system. **B**: The black and white and corresponding pseudo-color images of *T*_1_-weighted MR images for Gd-HPMA macrochelates observed by a 7.0 T MRI scanner. **C**, **D**: ΔSNR of the MR images **A**, **B** for Gd-HPMA macrochelates with various *C*_Gd_ observed at 3.0 **C** or 7.0 T D

**Fig. 2:**
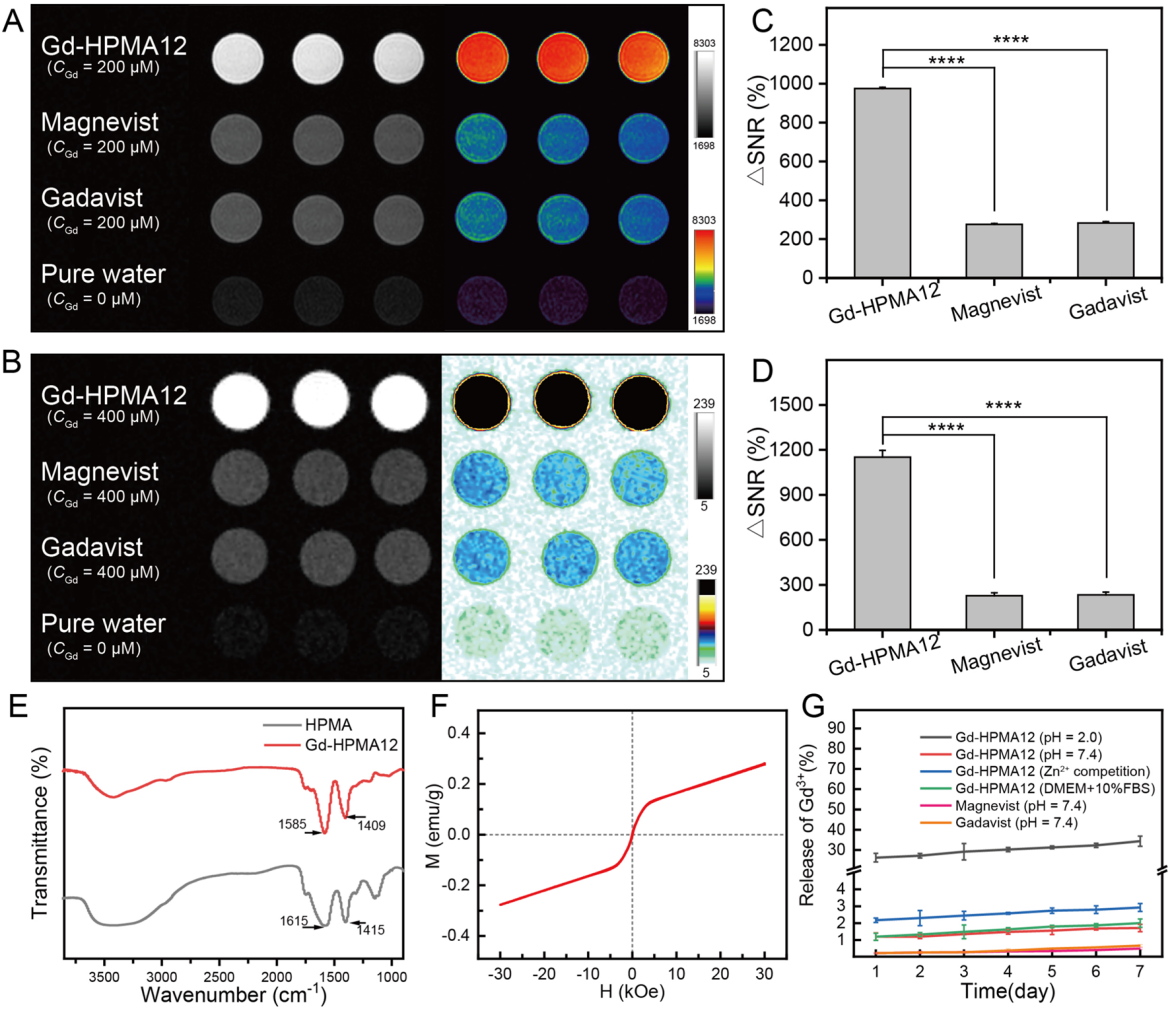
*T*_1_-weighted MRI performance and characterizations for Gd-HPMA macrochelates. **A**, **B**: The black & white and corresponding pseudo-color *T*_1_-weighted MR images of Gd-HPMA12 macrochelate atd 3.0 T **A** or 7.0 T **B** compared with Magnevist^®^, Gadavist^®^ and pure water. **C**, **D**: ΔSNR of the MR images **A**, **B** for Gd-HPMA12 compared with Magnevist^®^ or Gadavist^®^ at 3.0 T **C** or 7.0 T **D**. ****P < 0.0001. **E**: FT-IR spectra of HPMA and Gd-HPMA12. **F**: Field-dependent magnetization curve of Gd-HPMA12 measured by physical property measurement system (PPMS) at 300 K. **G**: The time-dependent release of free Gd^3+^ from Magnevist^®^, Gadavist^®^, Gd-HPMA12 in DMEM with 10% FBS, or saline solution of pH 2.0, pH 7.0, or pH 7.0 with double amount of Zn^2+^ compared with Gd in Gd-HPMA12

Figure 1A, B show the *T*_1_-weighted MR images of Gd-HPMA10-13, Magnevist^®^ and Gadavist^®^ with various *C*_Gd_ at 3.0 T and 7.0 T, indicating the feasibility of Gd-HPMA macrochelates for MR imaging. While signal intensities exhibit a progressive ascent commensurate with the augmenting *C*_Gd_ levels spanning from 6.25 to 200 μM, the intensity of Gadavist^®^ or Magnevist^®^ is much lower than that of Gd-HPMA10-13 macrochelates at equivalent Gd concentrations. The relaxivities are also reflected in the signal-to-noise ratio (SNR) of MR *T*_1_-weighted images, a metric that increases with raised the relaxivity and Gd concentration. The concentration gradient dependency of the MRI signal is further supported by the ΔSNR values in the 3.0 T and 7.0 T MRI images of Gd-HPMA10-13 (Fig. 1C, D). The black & white and corresponding pseudo-color *T*_1_-weighted MR images of Gd-HPMA12 macrochelate at 3.0 T (Fig. 2A) and 7.0 T (Fig. 2B) are presented for a more evident comparison with Magnevist^®^, Gadavist^®^ and pure water. Gd-HPMA12 achieves an ultrahigh ΔSNR value (975.2 ± 6.18%) compared with 272.4 ± 4.41% of Magnevist^®^ or 283.0 ± 6.98% of Gadavist^®^ at *C*_Gd_ = 200 μM (****P < 0.0001, 3.0 T, Fig. 2C), exhibiting the incredible performance in* T*_1_-weighted imaging. Similarly, the ΔSNR value also indicates a significant difference in Gd-HPMA12 (1152.3 ± 44.29%) compared to 228.5 ± 19.49% of Magnevist® or 233.9 ± 18.66% of Gadavist^®^ at *C*_Gd_ = 400 μM (****P < 0.0001, 7.0 T, Fig. 2D). The extremely high ΔSNR values at both 3.0 T and 7.0 T demonstrate that Gd-HPMA12 macrochelate has superior *T*_1_-weighted MRI performance compared with commercial GBCAs.

### Characterization of Gd-HPMA macrochelates

The composition and functional group of Gd-HPMA12 are demonstrated by the fourier transform infrared (FT-IR) absorption spectra (Fig. 2E). The FT-IR absorption spectrum of Gd-HPMA12 macrochelate shows characteristic peaks of C = O at 1615 cm^−1^ (antisymmetric) and 1415 cm^−1^ (symmetric), and C-O at 1140 cm^−1^, thus confirming the successful coordination of Gd^3+^ and carboxyl groups from HPMA. The C = O stretch vibrations include symmetric and antisymmetric stretch vibrations, which are slightly shifted compared with those of HPMA due to the formation of coordination bonds between -COOH and Gd^3+^ [32]. The similar shift of C = O stretching vibration has also been observed in Gd complexes with -COOH groups [33, 34].

The field-dependent magnetization curve (Fig. 2F) shows that Gd-HPMA12 macrochelate has a weak paramagnetism (*i.e*., zero coercivity, no hysteresis, and zero remanence in the M-H curve), whose saturation magnetization (M_s_) value is very low, only 0.218 emu/g at 30 kOe. According to the SBM relaxation mechanism [21], Gd^3+^ has seven unpaired electrons distributed evenly among the f-orbital, which results in a magnetic dipole moment independent of molecular orientation in an external magnetic field. This magnetic dipole moment causes Gd^3+^ to produce large paramagnetic relaxation enhancement along with absolutely no pseudocontact shift and generate paramagnetic agents with long electronic relaxation times (> 10^–8^ s) at field strengths > 3.0 T [35]. The weak paramagnetism is consistent with this theory because Gd^3+^ is used as a paramagnetic center to form the Gd-HPMA12 macrochelate.

As shown in Additional file 1: Figure S7, Gd-HPMA12 macrochelate has a negative charge (− 31.6 mV) due to the carboxyl groups of HPMA. Conventionally, cationic charges tend to induce opsonization and rapid clearance from the bloodstream, while a negative potential prevents the nonspecific uptake of Gd-HPMA12 macrochelate by normal cells during blood circulation thereby enhancing its passive targeting of tumors [36].

In Fig. 2G, the release behavior of Gd^3+^ in DMEM with 10% FBS (similar to plasma with many different components), or saline solution with pH 2.0, pH 7.0, or pH 7.0 with endogenous metal ions Zn^2+^ was explored. In DMEM with 10% FBS or saline solution with a pH of 7.0, the release of Gd^3+^ is less than 2%, indicating good stability of Gd-HPMA12. In saline solution with a pH of 2.0, the release of Gd^3+^ reaches up to 34% within 1 week, demonstrating instability of Gd-HPMA12 under strong acidic conditions. This conclusion aligns with the low Gd yields reported in Table S1. The competitive coordination between endogenous metal ions Zn^2+^ and Gd^3+^ was further investigated. Leak of ~ 2% Gd in the transmetallation experiment (in saline containing Zn^2+^) indicates the good stability of our Gd-HPMA12 macrochelate. Especially, the stability of Gd-HPMA12 is very similar to the clinically used GBCAs (*i.e.*, Magnevist^®^ and Gadavist^®^) as shown in Fig. 2G. The good stability of Gd-HPMA12 can be attributed to the excessive -COOH in HPMA and the robust formation of coordination bonds between HPMA and Gd^3+^.

### Efficacy and safety evaluation of the Gd-HPMA12 macrochelate on cells

**Fig. 3:**
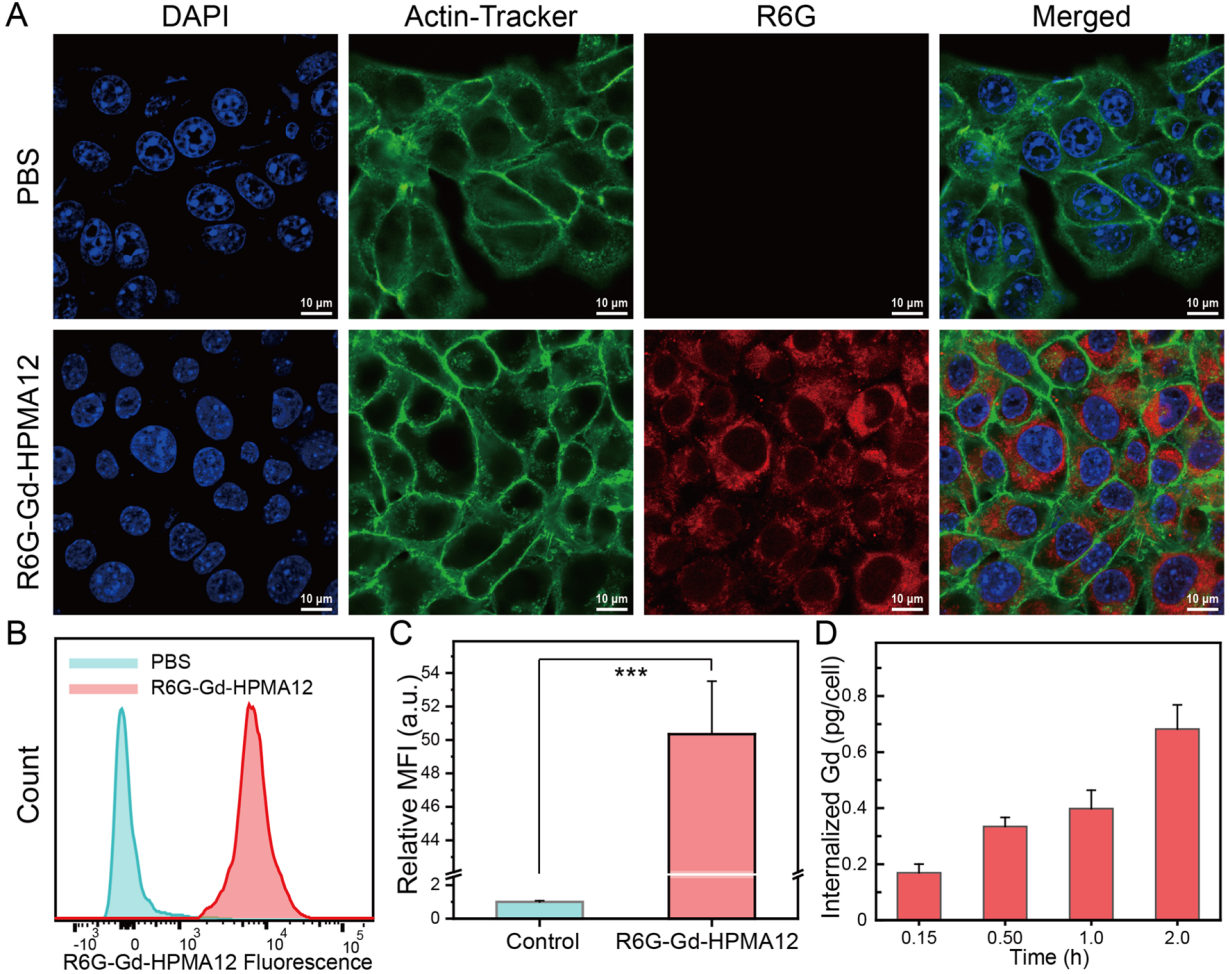
Evaluation of the efficacy and safety of Gd-HPMA12 on cells. **A**: CLSM images of 4T1 cells treated with R6G-Gd-HPMA12 for 2.0 h at 37 °C. PBS was used as a control. Green fluorescence: Actin-Tracker for cytoskeleton; red fluorescence: R6G for Gd-HPMA12; blue fluorescence: DAPI for nuclei. **B**, **C**: R6G fluorescence distributions of 4T1 cells incubated wit PBS or R6G-Gd-HPMA12 **B**, and the corresponding quantitative analysis **C** determined by flow cytometry, showing the level of cellular internalization of the R6G-loaded nanoparticles. Mean ± SD, *n* = 3. *** P < 0.001. **D**: The internalized amount of Gd-HPMA12 into 4T1 cells for various durations (0.25, 0.50, 1.0, or 2.0 h) measured by ICP

The cellular uptake of Gd-HPMA12 macrochelate was evaluated on 4T1 cells via laser scanning confocal microscopy (LSCM), flow cytometry, inductively coupled plasma mass spectrometry (ICP-MS) and MRI. As shown in Fig. 3A, 4T1 cells incubated with Rhodamine 6G (R6G)-labeled Gd-HPMA12 for 2.0 h at 37 °C exhibit a significant amount of red fluorescence inside their cell membranes. Usually, R6G is a small molecule fluorescent probe that can penetrate the cell nucleus. However, no red fluorescence is observed in the nucleus because unloaded R6G is absent in the R6G-Gd-HPMA12 solution. This observation is substantiated by the analysis of fluorescence intensity via flow cytometry (Fig. 3B). Importantly, there exists a statistically significant discrepancy in fluorescence intensity between the R6G-Gd-HPMA12 and PBS groups (*** P < 0.001, Fig. 3C), indicating the effective endocytosis of Gd-HPMA12. Furthermore, the quantitative evaluation of Gd-HPMA12 macrochelate internalization by 4T1 cells is subsequently determined using ICP-MS (Fig. 3D). After incubation for 0.15 h, 0.5 h, 1.0 h, or 2.0 h at 37 °C, Gd internalization in 4T1 cells increases continuously and reaches 0.68 ± 0.09 pg/cell at 2.0 h. Considering that Gd-HPMA12 macrochelate can be internalized in 4T1 cells, MRI of cancer cells in vitro (Additional file 1: Figure S8A) was utilized to assess the imaging and internalization capabilities of Gd-HPMA12 macrochelate. The intensity of *T*_1_-weighted MR images increases consistently with incubation time from 0 to 2 h, and the corresponding quantitative data shows significant differences in ΔSNR values between 0.25 h and 2 h (** P < 0.01, Figure S8B). All of these in vitro experimental results demonstrate that the 4T1 cells have an uptake ability for Gd-HPMA12 macrochelate. Gd-HPMA12 macromolecule has no specific target for 4T1 cells, and the uptake mechanism can be ascribed to passive endocytosis.

The biocompatibility of Gd-HPMA12 macrochelate on cells was evaluated by methyl thiazolyl tetrazolium (MTT) assay and hemocompatibility. The Gd-HPMA12 and Gadavist® exhibit good cell viability against 4T1 cells after being treated with various Gd concentrations ranging from 0 to 250 μg/mL, indicating a comparable biosafety between them (Additional file 1: Figure S9). The hemolysis ratios in vitro as shown in Additional file 1: Figure S10 further support the good biosafety of Gd-HPMA12 macrochelate, which are all less than 3.0% (Gd concentration = 0 ~ 500 μg/mL).

### In vivo biosafety evaluation and *T*_1_-weighted MR imaging

The biocompatibility in vivo was measured by blood routine analyses. On day 1, 7, or 21 post-injection (*i.v.*) of Gd-HPMA12 macrochelate to healthy female Balb/c mice, in vivo toxicity was assessed by blood routine analysis as shown in Figure S11, using the same volume of PBS as a control. The comparable blood routine indexes (*i.e.*, HCT, HGB, Lymph#, MCH, MCHC, MCV, MPV, PLT, Gran#, RBC and WBC) on day 1, 7, or 21 reveal that there are no alterations of the main hematic parameters on mice after administration of the Gd-HPMA12 macrochelate.

Excretion of Gd in urine or feces within 24 h after intravenous injection of Gd-HPMA12 (*C*_Gd_ = 5.0 mg/kg) was determined by ICP-MS (Additional file 1: Figure S12). At 0 ~ 8.0, 8.0 ~ 16 or 16 ~ 24 h, the Gd excretion in urine is respectively 70.70 ± 7.51, 17.52 ± 2.89 and 5.31 ± 2.65 I.D.%, while that in feces is lower than 2.55%. These results demonstrate that Gd-HPMA12 molecules are mainly eliminated via the kidney, and excreted out of the body within 24 h, resulting in the good biocompatibility with no acute and long-term toxicity, owing to the stable Gd^3+^ chelation with HPMA.

The blood circulation half-life (33.68 min) (Additional file 1: Figure S13) is a little bit longer than that of commercial GBCAs (~ 15 min) due to the larger molecular weight [27], providing more convenience (*i.e.*, a little bit longer processing time after administration) for patients and physicians. Clinically, the optimal time window for MRI (14–15 min) closely aligns with the half-life of commercial contrast agents (*i.e.*, GBCAs). This timeframe is somewhat constrained for MRI following the administration of GBCAs. The extended half-life of our Gd-HPMA12 (33.68 min) addresses the challenges associated with commercially available GBCAs.

**Fig. 4:**
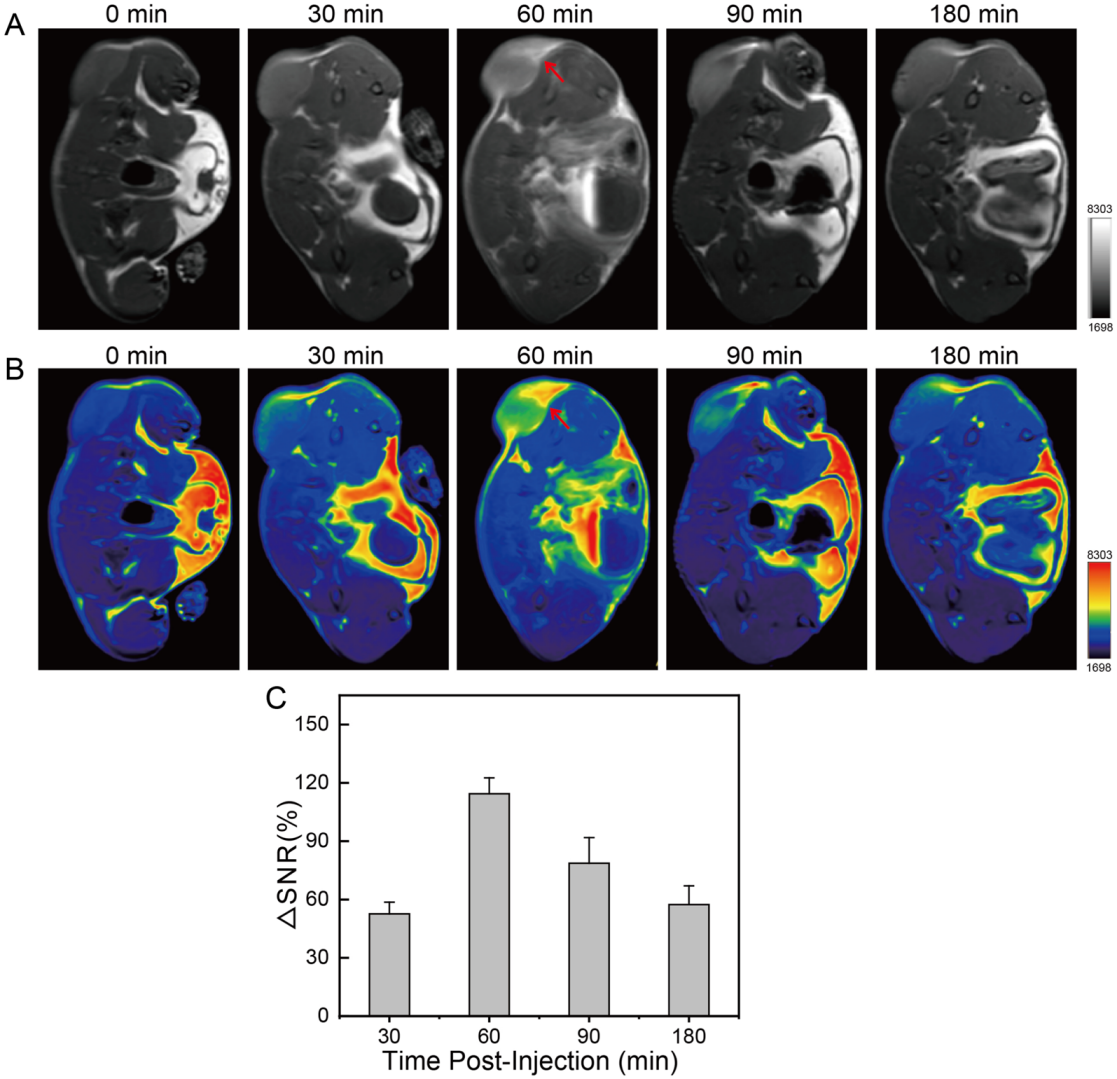
Evaluation of the efficacy and safety of Gd-HPMA12 on mice. A, B: *T*_1_-weighted MR images and the corresponding pseudo-color images of 4T1 tumor-bearing mice after intravenous injection of Gd-HPMA12 (*C*_Gd_ = 5.0 mg/kg) at different time intervals observed by a 3.0 T MRI scanner. **C**: ΔSNR for the MRI images **A**, **B** of tumors from 4T1 tumor-bearing mice post-injection of Gd-HPMA12 at different time intervals. Gd dosage = 5.0 mg/kg. Mean ± SD, *n* = 3

Encouraged by the MRI performance in aqueous solutions and biocompatibility, *T*_1_-weighted MR images and corresponding pseudo-color pictures (section direction: axial) of 4T1 tumor-bearing mice were observed on a 3.0 T MRI scanner at min 0, 30, 60, 90, or 180 min post-injection of Gd-HPMA12 macrochelate (Gd dose = 5.0 mg/kg) to validate the *T*_1_-weighted imaging capability in vivo. As shown in Fig. 4A, B, the intensity of MR images increases quickly and peaks at 60 min post-injection, with slight decrease until 180 min. Signal enhancement differences are expressed by ΔSNR to quantify signal changes in tumors at different time points (Fig. 4C), showing the maximum ΔSNR of 117 ± 18% for tumors in Balb/c female mice.

The biodistribution of Gd level in major organs and tumors of mice at 1.0 h or 12 h post-injection of Gd-HPMA12 (Gd dosage = 5.0 mg/kg) was determined by ICP-MS (Additional file 1: Figure S14). The high accumulation of Gd-HPMA12 macrochelate in tumors (*e.g.*, Gd content is 4.28 ± 0.68% ID/g at 1.0 h post-injection) is also one of the important reasons for the high tumor *T*_1_ MRI performance. Meanwhile the extremely low Gd levels at 12 h post-injection suggest that there is almost no Gd deposition in brain or bones.

Furthermore, the histological analysis (H and E staining) images of major organs (*i.e.*, heart, liver, spleen, lung, and kidney, Additional file 1: Figure S15) at days 2.0 post-injection of Gd-HPMA12 (*C*_Gd_ = 10.0 mg/kg) reveal no significant histological abnormalities or biological toxicity.

All of these in vivo experimental results suggest that Gd-HPMA exhibits fabulous *T*_1_-weighted MRI performance and excellent biocompatibility in mice.

### Large scale synthesis of Gd-HPMA macrochelates

**Fig. 5:**
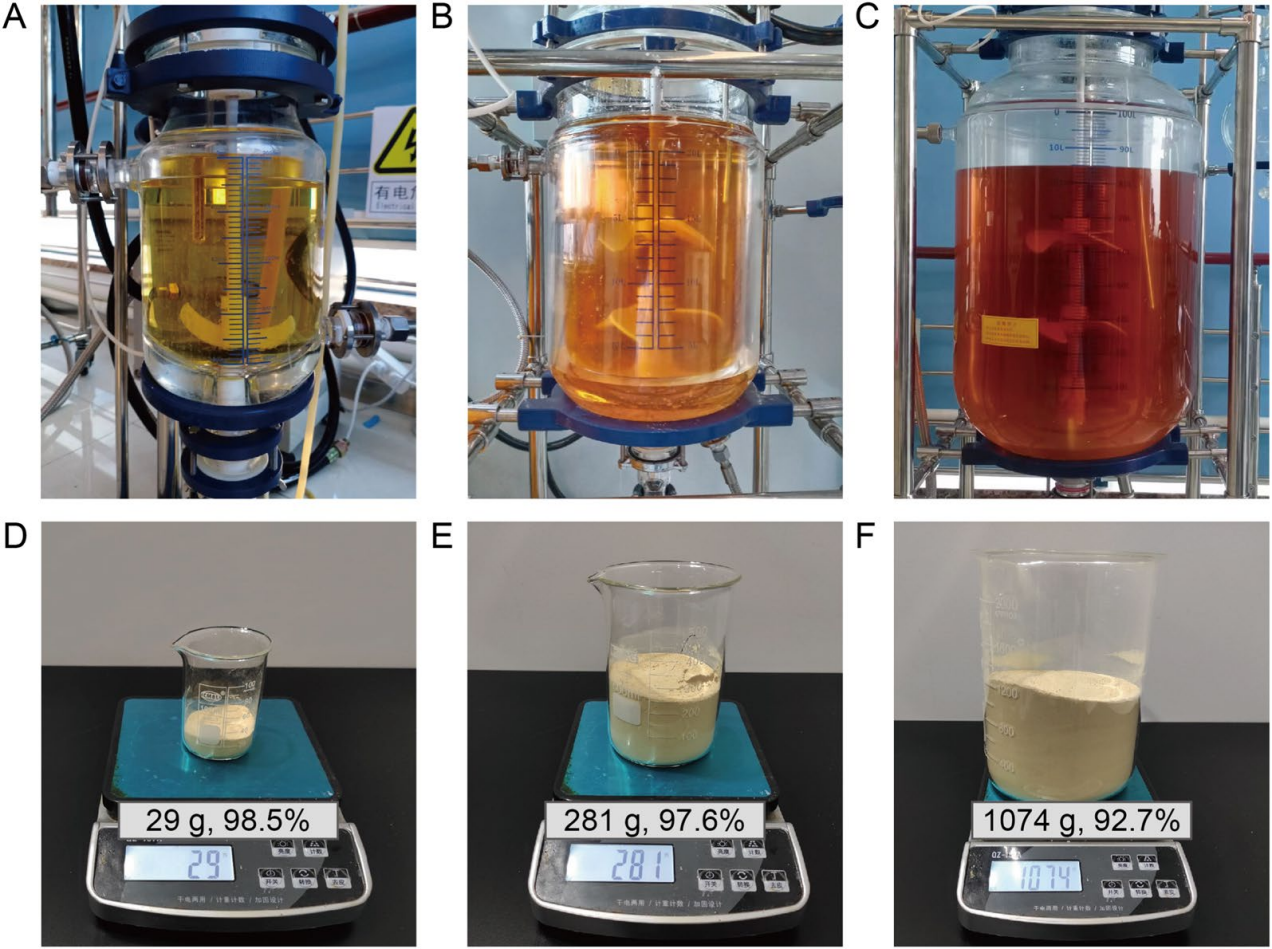
Large scale synthesis of the Gd-HPMAs. **A**–**C**: Photographs of 2.0 L **A**, 20 L **B**, or 100 L **C** reactors for large scale synthesis of the Gd-HPMAs. **D**–**F**: Weights and Gd yield of Gd-HPMA24 **D**, Gd-HPMA28 **E**, or Gd-HPMA29 **F** synthesized in 2.0 L **A**, 20 L **B** or 100 L **C** of reactor after freeze-drying

Large scale synthesis was further conducted for the Gd-HPMA macrochelates. 2.0, 20 and 100 L of reactors are shown in Fig. 5A–C for large scale facile synthesis of Gd-HPMA macrochelates. Referring to the synthesis conditions in a 20 mL of round bottom flask for Gd-HPMA12, the large scale synthesis conditions were optimized in the following five aspects, *i.e.*, pH value of the feeding HPMA solution, reaction temperature, concentrations of HPMA and GdCl_3_, the addition way of GdCl_3_ solution, and mechanical stirring rates. Additional file 1: Figure S16 shows the linear fitting of the 1/*T*_1_ or 1/*T*_2_ relaxation rate at 7.0 T as a function of *C*_Gd_ for Gd-HPMA14-29. The synthesis conditions and corresponding *r*_1_ and *r*_2_ values obtained from the slopes are summarized in Additional file 1: Table S3. The superhigh *r*_1_ values and ultralow *r*_2_/*r*_1_ ratios of Gd-HPMA14-29 result in the very high MRI performance (Additional file 1: Figures S17, S18).

Gd-HPMA16 with a high Gd yield shows a higher *r*_1_ value and similar *r*_2_/*r*_1_ ratio compared with Gd-HPMA14, 15 and 17, which indicates that pH 9.0 is the optimal condition. Besides, both Gd yield and *r*_1_ value decrease greatly with the decrease of reaction temperature, which means that low temperatures adversely affect the large-scale synthesis and 100 °C is the best reaction temperature. That’s because the high reaction temperature is conducive to the transferring of reactants and heat for large-scale synthesis.

10 mg/mL and 312.5 mM of concentrations are chosen as the optimal conditions for the feeding HPMA and GdCl_3_ due to the highest *r*_1_ value (15.66 mM^−1^ s^−1^) and similar *r*_2_/*r*_1_ ratio (3.21) at 7.0 T (Additional file 1: Table S3). The addition way of the feeding GdCl_3_ solution makes a very big difference for the relaxivity, *i.e.*, the *r*_2_ value and *r*_2_/*r*_1_ ratio decrease greatly when the feeding GdCl_3_ solution was drizzled into the reaction systems.

The optimal Gd-HPMA24 synthesized in a 2.0 L of reactor has a superhigh *r*_1_ value of 16.36 mM^−1^ s^−1^, an ultralow* r*_2_/*r*_1_ ratio of 2.08 at 7.0 T, a high yield of 98.5% (Additional file 1: Table S3), and a very high product weight of 29 g after freeze-drying (Fig. 5D).

20 L of reactor was further used to synthesize the Gd-HPMA25-29 macrochelates at 600, 500, 400 and 300 rpm of stirring. The comparable *r*_1_ values and *r*_2_/*r*_1_ ratios of Gd-HPMA25-29 macrochelates suggest that mechanical stirring rate has almost no influence on the MRI performance (Additional file 1: Table S3). 300 rpm was considered as the optimal mechanical stirring rate for 20 L synthesis because a low stirring rate may conserve electric energy. The optimal Gd-HPMA28 synthesized in a 20 L of reactor gains an awesome product weight (281 g) after freeze-drying (Fig. 5E), whose *r*_1_ value is 16.27 mM^−1^ s^−1^ and* r*_2_/*r*_1_ ratio is 2.09 at 7.0 T (Additional file 1: Table S3).

Finally, a larger scale synthesis was validated in a 100 L of reactor. The obtained Gd-HPMA29 with *r*_1_ value of 16.35 mM^−1^ s^−1^ and* r*_2_/*r*_1_ ratio of 2.05 at 7.0 T (Additional file 1: Table S3) has a high Gd yield of 92.7% and a high product weight (1074 g, Fig. 5F), which demonstrates the feasibility of kilogram scale facile synthesis for the Gd-HPMA macrochelates.

### Formulation optimization and sterilization at a high temperature

Referring to the specifications and physicochemical properties of commercially available drugs (*i.e.*, pH value, viscosity, osmolality, and density, Additional file 1: Table S4), the formulation optimization and sterilization at a high temperature was investigated for the Gd-HPMA29 macrochelate. A non-sodium amino buffer was considered as an excipient to reduce the viscosity of Gd-HPMA macrochelate at high concentrations [37, 38]. Meanwhile, dilute hydrochloric acid (HCl) was used to tune the pH. After optimization, the obtained Gd-HPMA30 formulation at high concentration (*C*_Gd_ = 100 mM) has a pH value of 7.97, osmolality of 691 mOsmol/kg water, density of 1.145 g/mL, and viscosity of 2.2 cP at 20 ℃ or 1.8 cP at 37 ℃ (Table S5), which meet all specifications and physicochemical criteria for clinical injections [26–28]. Moreover, the Gd-HPMA30 formulation has a high *r*_1_ value of 16.30 mM^−1^ s^−1^ and low* r*_2_/*r*_1_ ratio of 2.07 at 7.0 T (Additional file 1: Figures S19, S20, Table S5). The *r*_2_/*r*_1_ ratio of 2.07 at 7.0 T is much lower than that of our previously reported Gd-PAA macrochelate (4.2–5.5, 7.0 T), which benefits the *T*_1_ imaging. This result also demonstrates that excipients and sterilization at a high temperature have no adverse effect on the relaxation properties of Gd-HPMA30 macrochelate.

Finally, the biocompatibility in mice was further investigated by acute systemic toxicity. Median lethal dose (LD_50_) is commonly used to assess the acute systemic toxicity of drug preparations, which refers to the dose that causes 50% of death in tested animals [39, 40]. All mice were given one intravenous injection with Gd dosage of 5.00–150 mg/kg, and their mortalities are shown in Additional file 1: Table S6. After calculation, the laboratory mice exhibit a much higher LD_50_ of 479 mg/kg than free Gd^3+^(LD_50_ = 31 mg/kg) [41]. Gd-HPMA allows the most efficient MRI performance with an ultralow administration dosage because its *r*_1_ value at 3.0 T is 10 times higher than that of GBCAs. Referring to 15.7 mg/kg of administration dosage for GBCAs (Additional file 1: Table S5), 1.57 mg/kg is considered as an appropriate clinical usage dosage or median effective dose (ED_50_) for Gd-HPMA. The Gd-HPMA30 formulation reaches a therapeutic index (TI) of 305, which is much higher than 8–10 for iodine contrast agents or ~ 200 for GBCAs [42, 43]. Consequently, the physical characterizations, LD_50_ and TI for our Gd-HPMA30 formulation demonstrate the immense potential for clinical applications.

## Conclusions

In summary, in order to solve the problems of our previously reported Gd-PAA macrochelate, a superior macromolecule HPMA was found after screening macromolecules for Gd^3+^ coordination, generating Gd-HPMA macrochelate. The obtained Gd-HPMA12 macrochelate after synthesis conditions optimization achieves a very high *r*_1_ value (50.46 ± 1.01 mM^−1^ s^−1^, 3.0 T; 16.36 ± 0.87 mM^−1^ s^−1^, 7.0 T) and an ultralow *r*_2_/*r*_1_ ratio (1.21 ± 0.06, 3.0 T; 2.17 ± 0.12, 7.0 T), which are both very good for *T*_1_ imaging. The obtained Gd-HPMA29 synthesized in a 100 L of reactor has a *r*_1_ value of 16.35 mM^−1^ s^−1^ and* r*_2_/*r*_1_ ratio of 2.05 at 7.0 T, a high Gd yield of 92.7% and a high product weight of 1074 g, which demonstrates the feasibility of kilogram scale facile synthesis for the Gd-HPMA macrochelates. After investigation of the excipients, the obtained Gd-HPMA30 formulation has a pH value of 7.97, osmolality of 691 mOsmol/kg water, density of 1.145 g/mL and viscosity of 2.2 cP at 20 ℃ or 1.8 cP at 37 ℃, which meet all specifications and physicochemical criteria for clinical injections. The excipients and sterilization at a high temperature have no adverse effect on the relaxation properties of Gd-HPMA30 formulation (*r*_1_ = 16.30 mM^−1^ s^−1^, *r*_2_/*r*_1_ = 2.07, 7.0 T). The physical characterizations, LD_50_ and TI for the Gd-HPMA30 formulation demonstrate the potential for clinical applications.

### Supplementary Information

**Additional file 1: Table S1.** Synthesis conditions and characterization results of Gd-HPMAs. **Table S2.** Synthesis conditions and characterization results of Gd-HPMAs. **Table S3.** Large scale synthesis conditions and characterization results of Gd-HPMAs. **Table S4.** Specifications, dosages, and physicochemical properties for commercial contrast agents. **Table S5.** Physicochemical properties and characterization results for the Gd-HPMA30 formulation with adjuvants after high-temperature sterilization. **Table S6.** Acute systemic toxicity of the Gd-HPMA30 formulation after *i.v.* administration. **Fig. S1.** T_1_ relaxation rate plotted as a function of *C*_Gd_ for aqueous solutions of Gd-HPMA1-9 at 25 ℃ measured at 3.0 T. **Fig. S2.** *T*_2_ relaxation rate plotted as a function of *C*_Gd_ for aqueous solutions of Gd-HPMA1-9 at 25 ℃ measured at 3.0 T. **Fig. S3.** Influence of the Gd/HPMA molar ratio **A** or the pH value **B** on the *r*_1_ value and *r*_2_/*r*_1_ ratio. Mean ± SD, *n* = 3. **Fig. S4**. *T*_1_-weighted MR images of Gd-HPMA1-9 with various *C*_Gd_ (0 ~ 200 μM) observed by a 3.0 T clinical MRI system. **Fig. S5.** *T*_1_ **A–D** or *T*_2_ relaxation rate **E–H** plotted as a function of *C*_Gd_ for Gd-HPMA10-13 at 3.0 T. **Fig. S6.** *T*_1_ **A–D** or *T*_2_ relaxation rate **E–H** plotted as a function of *C*_Gd_ for Gd-HPMA10-13 at 7.0 T. **Fig. S7.** Zeta potential of Gd-HPMA12. **Fig. S8.** MRI of cancer cells in vitro. **Fig. S9.** Viabilities of 4T1 cells treated with Gd-HPMA12 compared with Gadavist^®^ in a Gd concentration range of 0–250 µg/mL. Mean ± SD, *n* = 3. **Fig. S10.** Hemolysis ratio induced by Gd-HPMA12 in a Gd concentration range of 0-500 µg/mL compared with pure water and PBS. Mean ± SD, n = 3. **Fig. S11.** Blood routine analyses of heathy mice at day 1.0, 7.0, or 21 post-injection (*i.v.*) of PBS, or Gd-HPMA12 (Gd dosage = 5.0 mg/kg). Mean ± SD, n = 3. The blood routine analyses include the following indicators: hematocrit (HCT), hemoglobin (HGB), lymphocyte count (Lymph#), mean corpusular hemoglobin (MCH), mean corpusular hemoglobin concerntration (MCHC), mean corpusular volume (MCV), mean platelet volume (MPV), platelet count (PLT), neutrophil ratio (Gran#), red blood cell (RBC), and white blood cell (WBC). **Fig. S12.** Excreted Gd content in urine or feces of healthy SD mice within 24 h after *i.v.* injection of Gd-HPMA12. Gd dosage = 5.0 mg/kg. Mean ± SD, *n* = 3. **Fig. S13.** Blood clearance profiles of Gd-HPMA12 in healthy Balb/c mice by tracking the Gd concentration in blood at different time intervals after *i.v.* injection (*n* = 3). **Fig. S14.** Biodistribution of Gd level in 4T1 tumor-bearing mice at 1.0 or 12 h post-injection of Gd-HPMA12 *via* tail vein. Gd dosage = 5.0 mg/kg. Mean ± SD, *n* = 3. **Fig.** **S15.** Histological analyses of main organs (H and E staining) obtained from healthy mice at day 2.0 post-injection (*i.v.*) of PBS, or Gd-HPMA12. **Fig. S16.** 1**/***T*_1_ or 1/*T*_2_ relaxation rate plotted as a function of *C*_Gd_ for Gd-HPMA14-29 at 7.0 T. **Fig. S17.** The black and white images of* T*_1_-weighted MR images of Gd-HPMA14-29 with various *C*_Gd_ (0 ~ 200 μM) observed by a 7.0 T clinical MRI system. **Fig. S18.** The pseudo-color images of *T*_1_-weighted MR images for Gd-HPMA14-29 with various *C*_Gd_ (0 ~ 200 μM) observed by a 7.0 T MRI scanner. **Fig. S19.** 1***/****T*_1_ or 1/*T*_2_ relaxation rate plotted as a function of *C*_Gd_ for Gd-HPMA30. Magnetic field = 7.0 T. **Fig. S20.** The black and white and corresponding pseudo-color images of *T*_1_-weighted MR images for Gd-HPMA30 macrochelate with various *C*_Gd_ (0 ~ 200 μM) observed by a 7.0 T MRI scanner.

## Data Availability

The data that support the findings of this study are available from the corresponding author upon reasonable request.

## Abbreviations

MRI: Magnetic resonance imaging
CAs: Contrast agents
PAA: Poly(acrylic acid)
HPMA: Hydrolyzed polymaleic anhydride
GBCAs: Gadolinium(III)-based contrast agents
FDA: Food and drug administration
NSF: Nephrogenic systemic fibrosis
CNS: Central nervous system
PRE: Paramagnetic relaxation enhancement
ICP-MS: Inductively coupled plasma mass spectrometry
MTT: Methyl thiazolyl tetrazolium
