# Supplementary material for: Kilogram scale facile synthesis and systematic characterization of a Gd-macrochelate as T1-weighted magnetic resonance imaging contrast agent
Source: J Nanobiotechnology. 2024 Apr 9;22:162. doi: 10.1186/s12951-024-02394-8 (PMC11005285; doi:10.1186/s12951-024-02394-8)
Supplement: Supplementary file 1 — Additional file 1: Table S1. Synthesis conditions and characterization results of Gd-HPMAs. Table S2. Synthesis conditions and characterization results of Gd-HPMAs. Table S3. Large scale synthesis conditions and characterization results of Gd-HPMAs. Table S4. Specifications, dosages, and physicochemical properties for commercial contrast agents. Table S5. Physicochemical properties and characterization results for the Gd-HPMA30 formulation with adjuvants after high-temperature sterilization. Table S6. Acute systemic toxicity of the Gd-HPMA30 formulation after i.v. administration. Fig. S1. T1 relaxation rate plotted as a function of CGd for aqueous solutions of Gd-HPMA1-9 at 25 ℃ measured at 3.0 T. Fig. S2. T2 relaxation rate plotted as a function of CGd for aqueous solutions of Gd-HPMA1-9 at 25 ℃ measured at 3.0 T. Fig. S3. Influence of the Gd/HPMA molar ratio A or the pH value B on the r1 value and r2/r1 ratio. Mean ± SD, n = 3. Fig. S4. T1-weighted MR images of Gd-HPMA1-9 with various CGd (0 ~ 200 μM) observed by a 3.0 T clinical MRI system. Fig. S5. T1 A–D or T2 relaxation rate E–H plotted as a function of CGd for Gd-HPMA10-13 at 3.0 T. Fig. S6. T1 A–D or T2 relaxation rate E–H plotted as a function of CGd for Gd-HPMA10-13 at 7.0 T. Fig. S7. Zeta potential of Gd-HPMA12. Fig. S8. MRI of cancer cells in vitro. Fig. S9. Viabilities of 4T1 cells treated with Gd-HPMA12 compared with Gadavist® in a Gd concentration range of 0–250 µg/mL. Mean ± SD, n = 3. Fig. S10. Hemolysis ratio induced by Gd-HPMA12 in a Gd concentration range of 0-500 µg/mL compared with pure water and PBS. Mean ± SD, n = 3. Fig. S11. Blood routine analyses of heathy mice at day 1.0, 7.0, or 21 post-injection (i.v.) of PBS, or Gd-HPMA12 (Gd dosage = 5.0 mg/kg). Mean ± SD, n = 3. The blood routine analyses include the following indicators: hematocrit (HCT), hemoglobin (HGB), lymphocyte count (Lymph#), mean corpusular hemoglobin (MCH), mean corpusular hemoglobin concerntration (MCHC), mean corpusular v [file 12951_2024_2394_MOESM1_ESM.doc]

**Additional file Information**

**Kilogram Scale Facile Synthesis and Systematic Characterization of a Gd-Macrochelate as T_1_-Weighted Magnetic Resonance Imaging Contrast Agent**

*Meng Shi, Wei Xiong,* Jie Feng, Lihe Wu, Jing Yang,* *Yudie Lu, Xuanyi Lu, Qingdeng Fan, Hemin Nie, Yunlu Dai, Chenggong Yan,* Ye Tian,* Zheyu Shen**

M. Shi, L. Wu, Dr. J. Yang, Y. Lu, X. Lu, Q. Fan, Prof. Y. Tian, Prof. Z. Shen

School of Biomedical Engineering, Southern Medical University, 1023 Shatai South Road, Baiyun, Guangzhou, Guangdong 510515, China.

E-mail: sz@smu.edu.cn (Z.S.), tymty@smu.edu.cn (Y.T.)

Dr. W. Xiong, Dr. J. Feng, Prof. C. Yan

Medical Imaging Center, Nanfang Hospital, Southern Medical University, 1023 Shatai South Road, Baiyun, Guangzhou, Guangdong 510515, China.

E-mail: ycgycg007@gmail.com (C.Y.), xiongwei@smu.edu.cn (W.X.)

Prof. H. Nie

Department of Biomedical Sciences, College of Biology, Hunan University, 52 Tianmu Road, Yuelu, Changsha, Hunan 410082, China.

Prof. Y. Dai

Faculty of Health Sciences and MoE Frontiers Science Center for Precision Oncology, University of Macau, Macau SAR, 999078, China.

**METHODS**

**Materials and reagents**

Hydrolyzed polymaleic anhydride (HPMA, average M_w_ ~ 1200 Da, 48 wt% in H_2_O) was purchased from Aladdin (Sichuan, China). Gadolinium(III) chloride hexahydrate (GdCl_3_·6H_2_O, 99%), rhodamine 6G (R6G) and sodium hydroxide (NaOH, 97%) were purchased from Macklin (Shanghai, China). Tromethamine (Tris) was purchased from Sigma-Aldrich (USA). Hydrochloric acid (HCl, 36%-38%) was Guangzhou Chemical Reagent Factory (Guangzhou, China). Thiazolyl blue tetrazolium bromide (MTT) was purchased from Shanghai Acmec Biochemical Co., Ltd. 2-(4-Amidinophenyl)-6-indolecarbamidine dihydrochloride (DAPI) and Phalloidin-FITC were purchased from Beyotime Biotechnology (Shanghai, China).

**Cell culture**

4T1 cells (mouse breast cancer cell line, provided by Southern Medical University) were cultured in the complete DMEM culture medium supplemented with 10% FBS, 100 U/mL of penicillin G sodium, and 100 µg/mL of streptomycin sulfate. All of the cells were incubated at 37 ^o^C in a humidified atmosphere containing 5.0% CO_2_.

**MRI performance *in vitro***

After membrane dialysis against Milli-Q water for three days, MRI performance of Gd-HPMAs was tested. The aqueous solutions of Gd-HPMAs were prepared with gradient *C*_Gd_, and then *T*_1_-weighted phantom images and relaxation times were acquired on a 7.0 T MRI scanner (Bruker, PharmaScan70/16 US) or a clinical 3.0 T MRI scanner system (3.0 T, Philips, Ingenia, NL) at 25 ℃. The *r*_1_ relaxivities were calculated from the slope of the linear fitting lines of 1/*T*_1_ versus *C*_Gd_. For a 7.0 T MRI scanner, the parameters for *T*_1_ measurements were set as follows: echo time (TE) = 7.3 ms, repetition time (TR) = 120 ms; the parameters for *T*_2_ measurements were set as follows: TE= 120 ms, TR = 5000 ms. For a 3.0 T MRI scanner, the *T*_1_ measurements sequence was: TE= 8.2 ms, TR = 200 ms; the parameters for *T*_2_ measurements were set as follows: TE= 80 ms, TR = 5000 ms. Meanwhile, MR images were analyzed by measuring signal intensity with the software Image J. The signal-to-noise ratio (SNR) and ΔSNR value (*i.e.*, signal enhancement) were calculated according to the following equations:

SNR = SI_mean_/SD_noise_

ΔSNR = (SNR_sample_ - SNR_control_)/SNR_control_ × 100%

**Tumor model**

All animal procedures were performed in accordance with the Guidelines for Care and Use of Laboratory Animals of Southern Medical University, and approved by the Animal Ethics Committee of Southern Medical University. The assigned approval/accreditation number is SYXK(YUE)2021-0167.

To establish xenograft tumor models, female nude Balb/c mice (five-week-old, 15-20 g) were subcutaneously implanted with 100 μL of 5.0 × 10^6^ 4T1 cells in the right back side. The size of tumors was measured every other day with a vernier caliper, and the tumor volumes were calculated as follows: tumor volume (mm^3^) = width^2^ × length / 2.

**Statistical analysis**

Statistical analysis of data was performed with Student’s t-test or one-way analysis of variance (ANOVA). Data were presented as mean ± SD. The level of significance was defined as *p < 0.05, **p < 0.01, ***p < 0.001, or ****p < 0.0001.

Additional file **Tables**

**Table S1.** Synthesis conditions and characterization results of Gd-HPMAs.

| Sample Nomenclature | *C*_HPMA_ *^a^*  (mg/mL) | *C*_GdCl3_ *^a^*  (mM) | pH | Gd/HPMA molar ratio *^b^* | COOH/Gd molar ratio *^c^* | T  (℃) | Gd Yield *^d^*  (%) | H_0_ (T) | *r*_1_  (mM^-1^ s^-1^) | *r*_2_  (mM^-1^ s^-1^) | *r*_2_ / *r*_1_ |
| --- | --- | --- | --- | --- | --- | --- | --- | --- | --- | --- | --- |
| Gd-HPMA1 | 4.0 | 62.5 | 7.0 | 0.38 | 56 | 100 | 90.3 | 3.0 | 47.57±0.92 | 56.91±1.14 | 1.20±0.03 |
| Gd-HPMA2 | 4.0 | 125 | 7.0 | 0.75 | 28 | 100 | 94.6 | 3.0 | 48.14±0.56 | 57.37±1.27 | 1.19±0.04 |
| Gd-HPMA3 | 4.0 | 250 | 7.0 | 1.50 | 14 | 100 | 95.6 | 3.0 | 47.67±0.81 | 57.66±1.73 | 1.21±0.06 |
| Gd-HPMA4 | 4.0 | 500 | 7.0 | 3.01 | 7.0 | 100 | 75.6 | 3.0 | 47.19±0.47 | 57.63±0.94 | 1.22±0.01 |
| Gd-HPMA5 | 4.0 | 125 | 5.0 | 0.75 | 28 | 100 | 70.8 | 3.0 | 46.99±0.91 | 55.05±1.18 | 1.17±0.05 |
| Gd-HPMA6 | 4.0 | 125 | 6.0 | 0.75 | 28 | 100 | 75.5 | 3.0 | 47.93±1.02 | 57.01±1.16 | 1.19±0.05 |
| Gd-HPMA7 | 4.0 | 125 | 8.0 | 0.75 | 28 | 100 | 89.6 | 3.0 | 48.04±1.06 | 57.64±2.03 | 1.20±0.07 |
| Gd-HPMA8 | 4.0 | 125 | 9.0 | 0.75 | 28 | 100 | 95.3 | 3.0 | 49.84±0.32 | 59.95±0.39 | 1.20±0.07 |
| Gd-HPMA9 | 4.0 | 125 | 10.0 | 0.75 | 28 | 100 | 90.9 | 3.0 | 48.55±0.52 | 58.67±1.25 | 1.21±0.03 |

*^a)^* Concentration of the feeding HPMA (M_w_ = 1200 Da) and GdCl_3_ before reaction.

*^b)^* Calculated from the molar ratio of Gd to HPMA in the feeding materials.

*^c)^* Calculated from the molar ratio of carboxyl group of HPMA to Gd in the feeding materials.

*^d)^* Calculated from the molar percentage of Gd in the obtained Gd-HPMA to that in the feeding materials.

**Table S2. Synthesis conditions and characterization results of Gd-HPMAs.**

| Sample Nomenclature | T  (℃) | Nitrogen *^a^* | Gd Yield *^b^*  (%) | H_0_ (T) | *r*_1_  (mM^-1^ s^-1^) | *r*_2_  (mM^-1^ s^-1^) | *r*_2_ / *r*_1_ |
| --- | --- | --- | --- | --- | --- | --- | --- |
| Gd-HPMA10 | 100 | No | 95.5 | 3.0 | 49.20±0.70 | 58.78±1.19 | 1.20±0.04 |
|  |  |  |  | 7.0 | 16.40±0.07 | 34.54±0.24 | 2.11±0.00 |
| Gd-HPMA11 | 100 | Yes | 97.9 | 3.0 | 49.49±0.59 | 59.89±0.91 | 1.21±0.01 |
|  |  |  |  | 7.0 | 16.44±0.20 | 33.85±0.24 | 2.06±0.12 |
| Gd-HPMA12 | 25 | No | 95.3 | 3.0 | 50.46±1.01 | 60.96±1.66 | 1.21±0.06 |
|  |  |  |  | 7.0 | 16.36±0.87 | 35.34±0.44 | 2.17±0.12 |
| Gd-HPMA13 | 25 | Yes | 94.8 | 3.0 | 49.81±0.51 | 60.21±0.39 | 1.21±0.02 |
|  |  |  |  | 7.0 | 16.31±0.45 | 35.74±1.43 | 2.20±0.11 |

*^a)^* The reactions were protected under nitrogen atmosphere or not.

*^b)^* Calculated from the molar percentage of Gd in the obtained Gd-HPMA to that in the feeding materials.

**Table S3.** Large scale synthesis conditions and characterization results of Gd-HPMAs.

| Sample Nomenclature | *C*_HPMA_ *^a^*  (mg/mL) | V_HPMA_  (L) | *C*_GdCl3_ *^a^*  (mM) | V_GdCl3_  (L) | pH | T  (℃) | Addition Method *^b^* | Rev *^c^*  (rpm) | Gd Yield *^d^* (%) | H_0_ (T) | *r*_1_  (mM^-1^ s^-1^) | *r*_2_  (mM^-1^ s^-1^) | *r*_2_ / *r*_1_ |
| --- | --- | --- | --- | --- | --- | --- | --- | --- | --- | --- | --- | --- | --- |
| Gd-HPMA14 | 4.0 | 2.0 | 125.0 | 0.04 | 7.0 | 100 | Pour | 600 | 81.5 | 7.0 | 14.44 | 59.27 | 4.11 |
| Gd-HPMA15 | 4.0 | 2.0 | 125.0 | 0.04 | 8.0 | 100 | Pour | 600 | 87.8 | 7.0 | 14.67 | 51.36 | 3.50 |
| Gd-HPMA16 | 4.0 | 2.0 | 125.0 | 0.04 | 9.0 | 100 | Pour | 600 | 90.2 | 7.0 | 15.14 | 52.63 | 3.48 |
| Gd-HPMA17 | 4.0 | 2.0 | 125.0 | 0.04 | 10.0 | 100 | Pour | 600 | 90.8 | 7.0 | 14.64 | 49.56 | 3.39 |
| Gd-HPMA18 | 4.0 | 2.0 | 125.0 | 0.04 | 9.0 | 80.0 | Pour | 600 | 89.6 | 7.0 | 14.51 | 50.24 | 3.46 |
| Gd-HPMA19 | 4.0 | 2.0 | 125.0 | 0.04 | 9.0 | 60.0 | Pour | 600 | 74.1 | 7.0 | 13.10 | 52.63 | 4.02 |
| Gd-HPMA20 | 4.0 | 2.0 | 125.0 | 0.04 | 9.0 | RT | Pour | 600 | 34.5 | 7.0 | 12.72 | 50.13 | 3.94 |
| Gd-HPMA21 | 8.0 | 2.0 | 250.0 | 0.04 | 9.0 | 100 | Pour | 600 | 92.3 | 7.0 | 15.20 | 48.55 | 3.19 |
| Gd-HPMA22 | 10 | 2.0 | 312.5 | 0.04 | 9.0 | 100 | Pour | 600 | 94.6 | 7.0 | 15.66 | 50.24 | 3.21 |
| Gd-HPMA23 | 12 | 2.0 | 375.0 | 0.04 | 9.0 | 100 | Pour | 600 | 50.6 | 7.0 | 15.28 | 51.00 | 3.34 |
| Gd-HPMA24 | 10 | 2.0 | 312.5 | 0.04 | 9.0 | 100 | Drizzle | 600 | 98.5 | 7.0 | 16.36 | 34.00 | 2.08 |
| Gd-HPMA25 | 10 | 20 | 312.5 | 0.40 | 9.0 | 100 | Drizzle | 600 | 98.9 | 7.0 | 16.09 | 33.97 | 2.11 |
| Gd-HPMA26 | 10 | 20 | 312.5 | 0.40 | 9.0 | 100 | Drizzle | 500 | 97.6 | 7.0 | 16.26 | 33.98 | 2.09 |
| Gd-HPMA27 | 10 | 20 | 312.5 | 0.40 | 9.0 | 100 | Drizzle | 400 | 99.1 | 7.0 | 16.33 | 33.51 | 2.05 |
| Gd-HPMA28 | 10 | 20 | 312.5 | 0.40 | 9.0 | 100 | Drizzle | 300 | 97.6 | 7.0 | 16.27 | 34.04 | 2.09 |
| Gd-HPMA29 | 10 | 80 | 312.5 | 1.60 | 9.0 | 100 | Drizzle | 300 | 92.7 | 7.0 | 16.35 | 33.52 | 2.05 |

*^a)^* Concentration of the feeding HPMA (M_w_ = 1200 Da) and GdCl_3_ before reaction.

*^b)^* The GdCl_3_ solution was poured or drizzled into the reaction systems.

*^c)^* Revolutions per minute (rpm), the speed of mechanical stirring in the reactions.

*^d)^* Calculated from the molar percentage of Gd in the obtained Gd-HPMA to that in the feeding materials.

**Table S4.** Specifications, dosages, and physicochemical properties for commercial contrast agents [1-3].

| Trade Name | Specification *^a^* | Dosage of Gd  (mg/kg) | pH Value | Osmolality (mOsmol/kg) | Viscosity  (cP) | | Density  (g/mL) |
| --- | --- | --- | --- | --- | --- | --- | --- |
|  |  |  |  |  | 20℃ | 37℃ |  |
| Magnevist® | 0.5 M | 15.7 | 6.5-8.0 | 1960 | 4.9 | 2.9 | 1.195 |
| Gadavist® | 1.0 M | 15.7 | 6.5-8.0 | 1603 | - | 5.0 | 1.300 |
| Dotarem® | 0.5 M | 15.7 | 6.5-8.0 | 1350 | 3.4 | 2.0 | 1.175 |
| ProHance® | 0.5 M | 15.7 | 6.5-8.0 | 630 | 2.0 | 1.3 | 1.137 |

*^a)^* Concentration of commercial contrast agents.

**References**

[1] Hao D, Ai T, Goerner F, Hu X, Runge VM, Tweedle M. MRI contrast agents: basic chemistry and safety. JMRI 2012, 36, 1060-1071.

[2] Lin SP, Brown JJ. MR contrast agents: physical and pharmacologic basics. JMRI 2007, 25, 884-899.

[3] Laurent S, Elst LV, Muller RN. Comparative study of the physicochemical properties of six clinical low molecular weight gadolinium contrast agents. Contrast Media Mol. Imaging 2006, 1, 128-137.

**Table S5.** Physicochemical properties and characterization results for the Gd-HPMA30 formulation with adjuvants after high-temperature sterilization.

| Sample Nomenclature | *C_Gd_*  (mM) | pH | Osmolality (mOsmol/kg) | Viscosity  (cP) | | Density  (g/mL) | H_0_(T) | *r*_1_  (mM^-1^ s^-1^) | *r*_2_  (mM^-1^ s^-1^) | *r*_2_ / *r*_1_ |
| --- | --- | --- | --- | --- | --- | --- | --- | --- | --- | --- |
|  |  |  |  | 20℃ | 37℃ |  |  |  |  |  |
| Gd-HPMA30 | 100 | 7.97 | 691 | 2.2 | 1.8 | 1.145 | 7.0 | 16.30 | 33.81 | 2.07 |

**Table S6.** Acute systemic toxicity of the Gd-HPMA30 formulation after *i.v.* administration.

| Group Number *^a^* | Dosage  (mg/kg) | Total Number of Mice Dosed | Number of Survival Mice | Mortality  (%) |
| --- | --- | --- | --- | --- |
| 1 | 150 | 10 | 9 | 10 |
| 2 | 75.0 | 10 | 10 | 0 |
| 3 | 30.0 | 10 | 10 | 0 |
| 4 | 12.0 | 10 | 10 | 0 |
| 5 | 5.00 | 10 | 10 | 0 |

*^a )^*50 SD mice (body weight = 198.4 ± 0.7 g) were divided into 5 groups randomly with 10 mice in each group, including 5 males and 5 females.

Additional file **Figures**


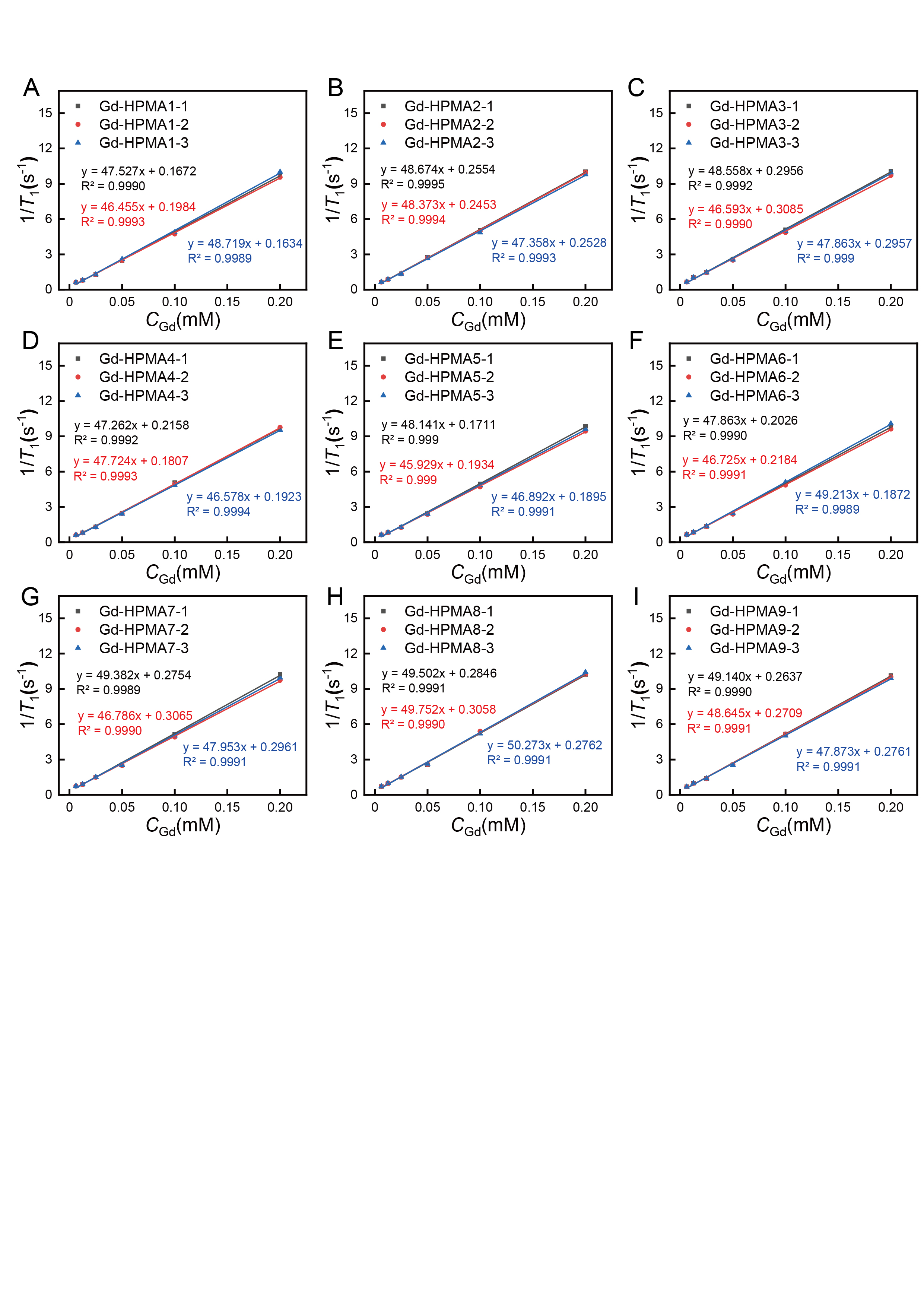


**Fig. S1**. *T*_1_ relaxation rate plotted as a function of *C*_Gd_ for aqueous solutions of Gd-HPMA1-9 at 25 ℃ measured at 3.0 T.


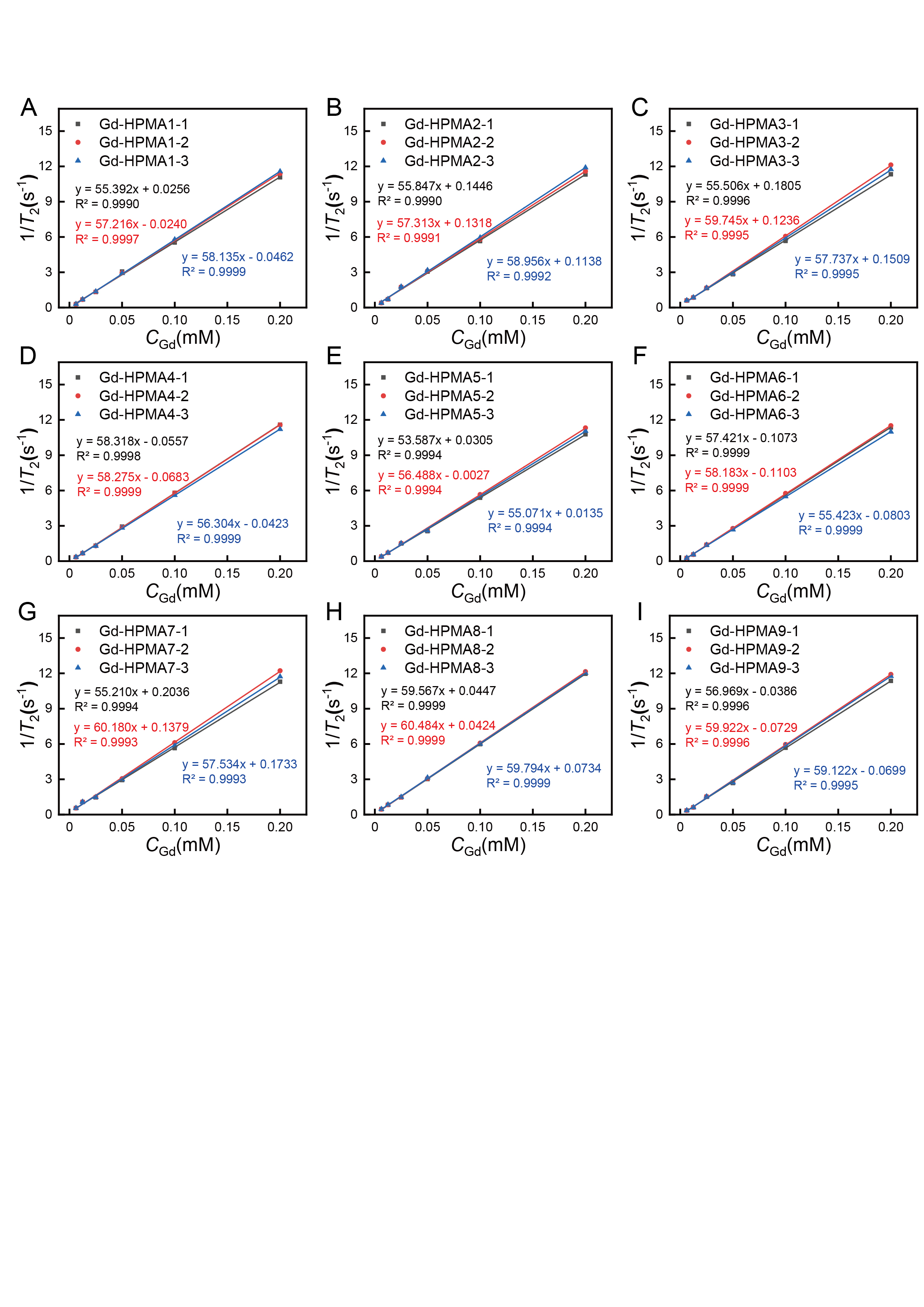


**Fig. S2**. *T*_2_ relaxation rate plotted as a function of *C*_Gd_ for aqueous solutions of Gd-HPMA1-9 at 25 ℃ measured at 3.0 T.


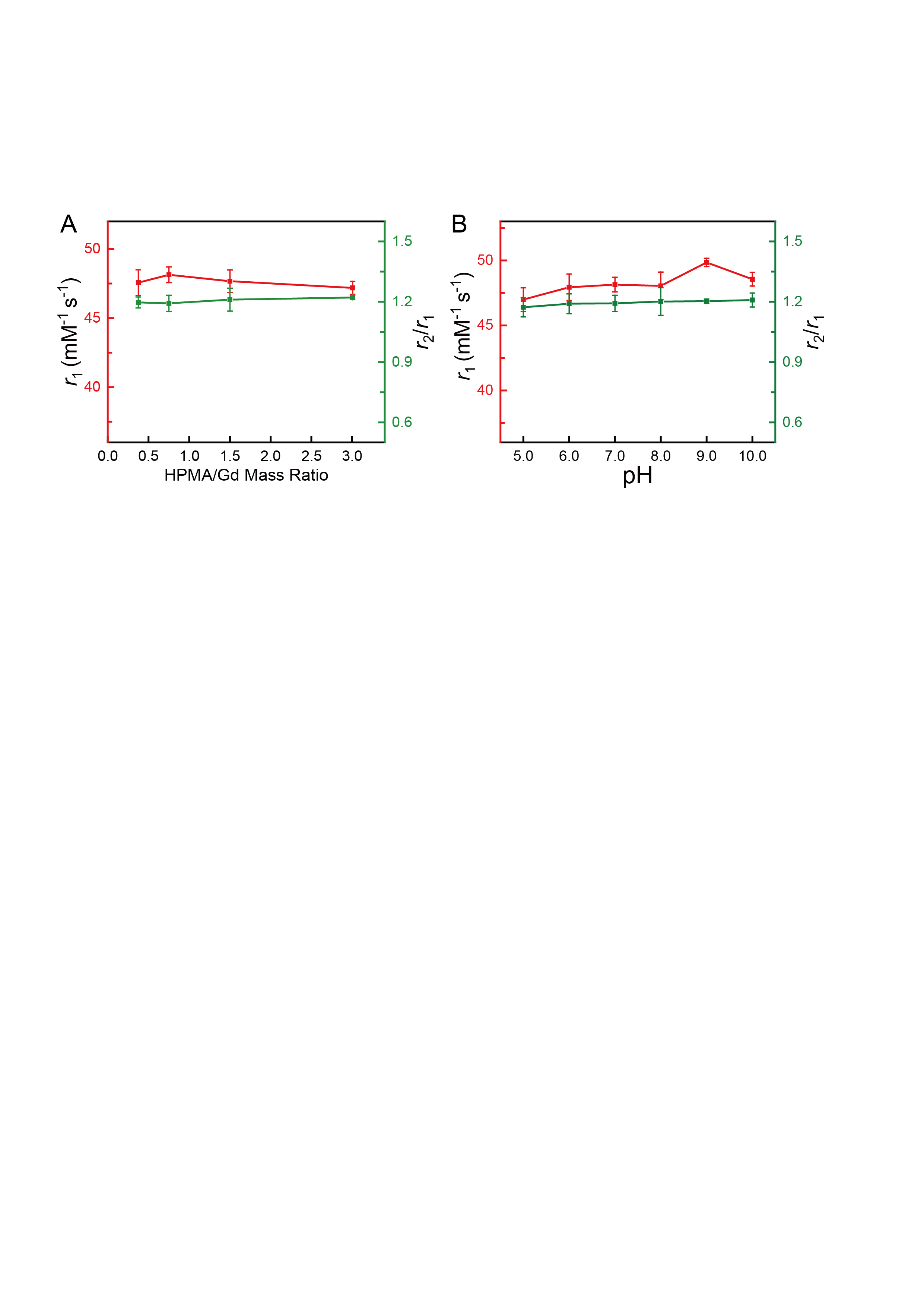


**Fig. S3**. Influence of the Gd/HPMA molar ratio (A) or the pH value (B) on the *r*_1_ value and *r*_2_/*r*_1_ ratio. Mean ± SD, *n* = 3.


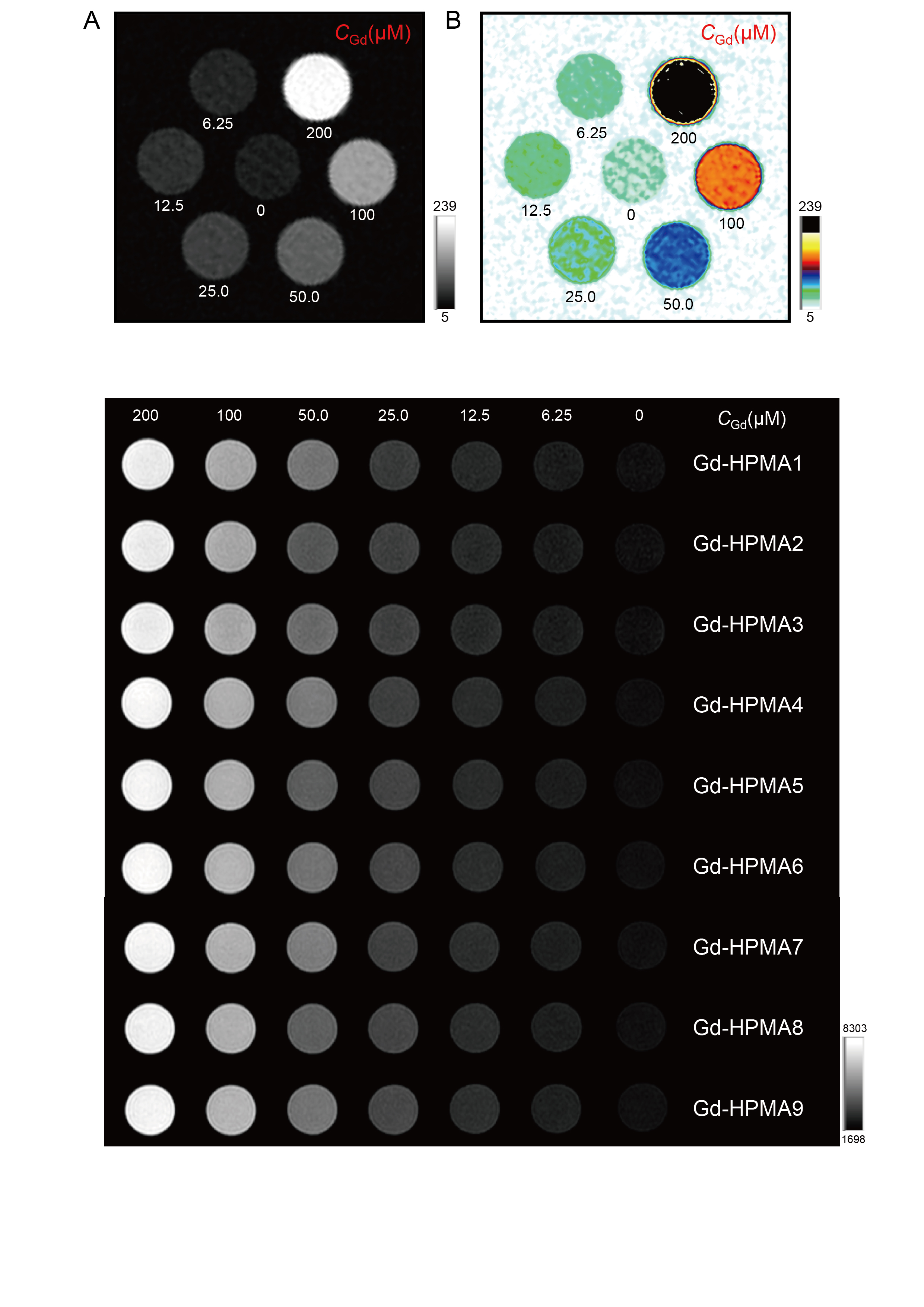


**Fig. S4**. *T*_1_-weighted MR images of Gd-HPMA1-9 with various *C*_Gd_ (0 ~ 200 μM) observed by a 3.0 T clinical MRI system.


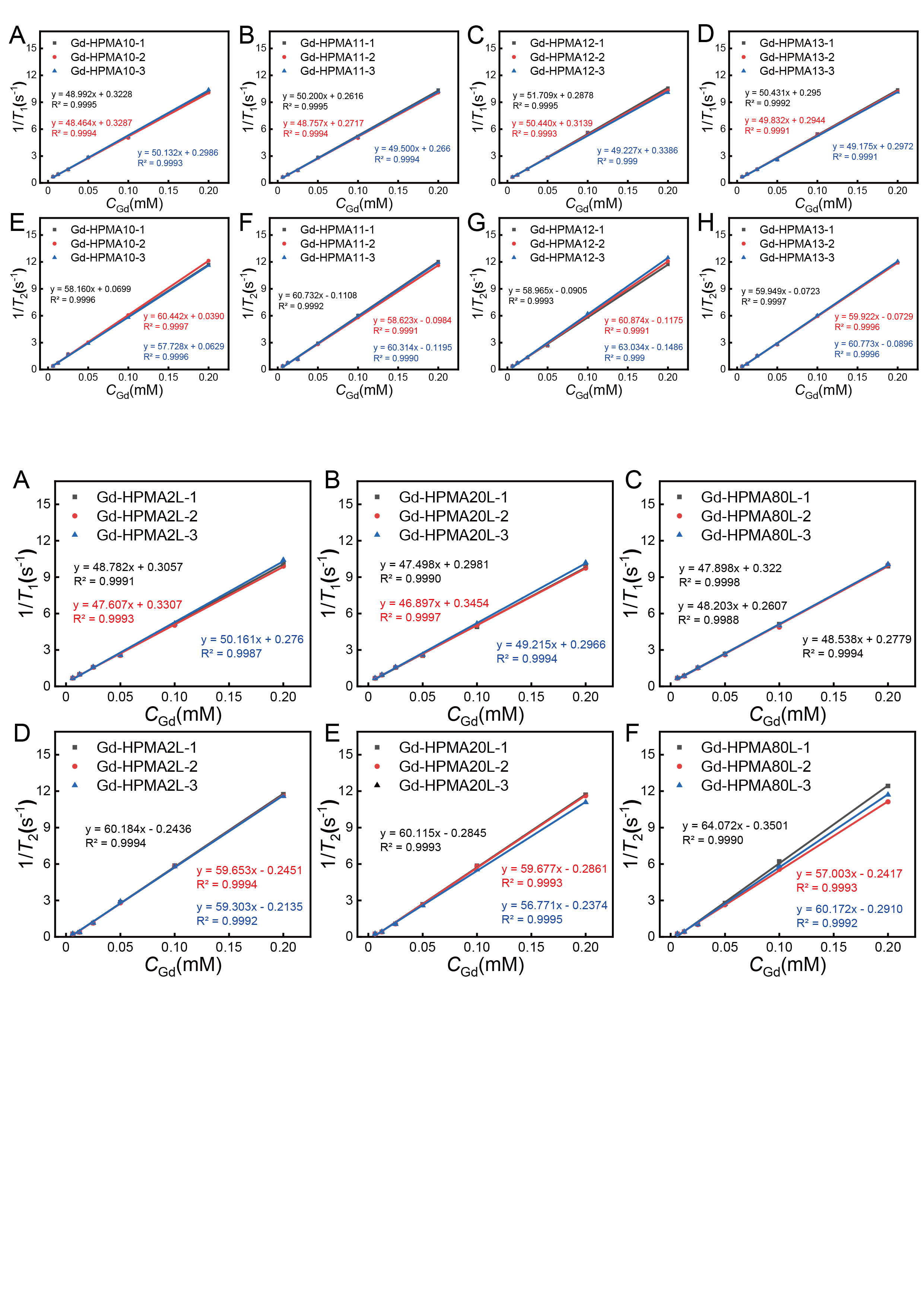


**Fig. S5.** *T*_1_ (**A-D**) or *T*_2_ relaxation rate (**E-H**) plotted as a function of *C*_Gd_ for Gd-HPMA10-13 at 3.0 T.


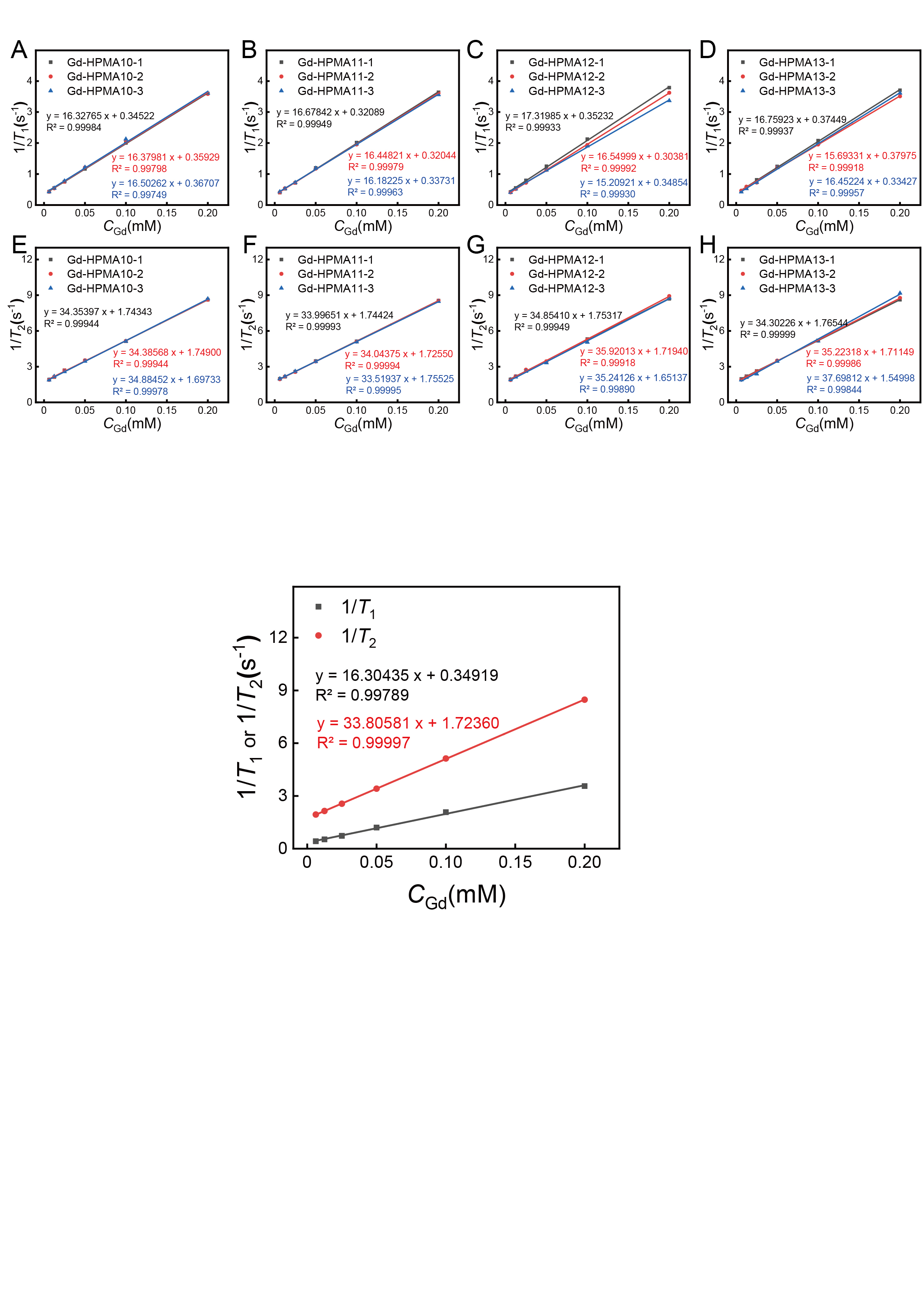


**Fig. S6.** *T*_1_ (**A-D**) or *T*_2_ relaxation rate (**E-H**) plotted as a function of *C*_Gd_ for Gd-HPMA10-13 at 7.0 T.


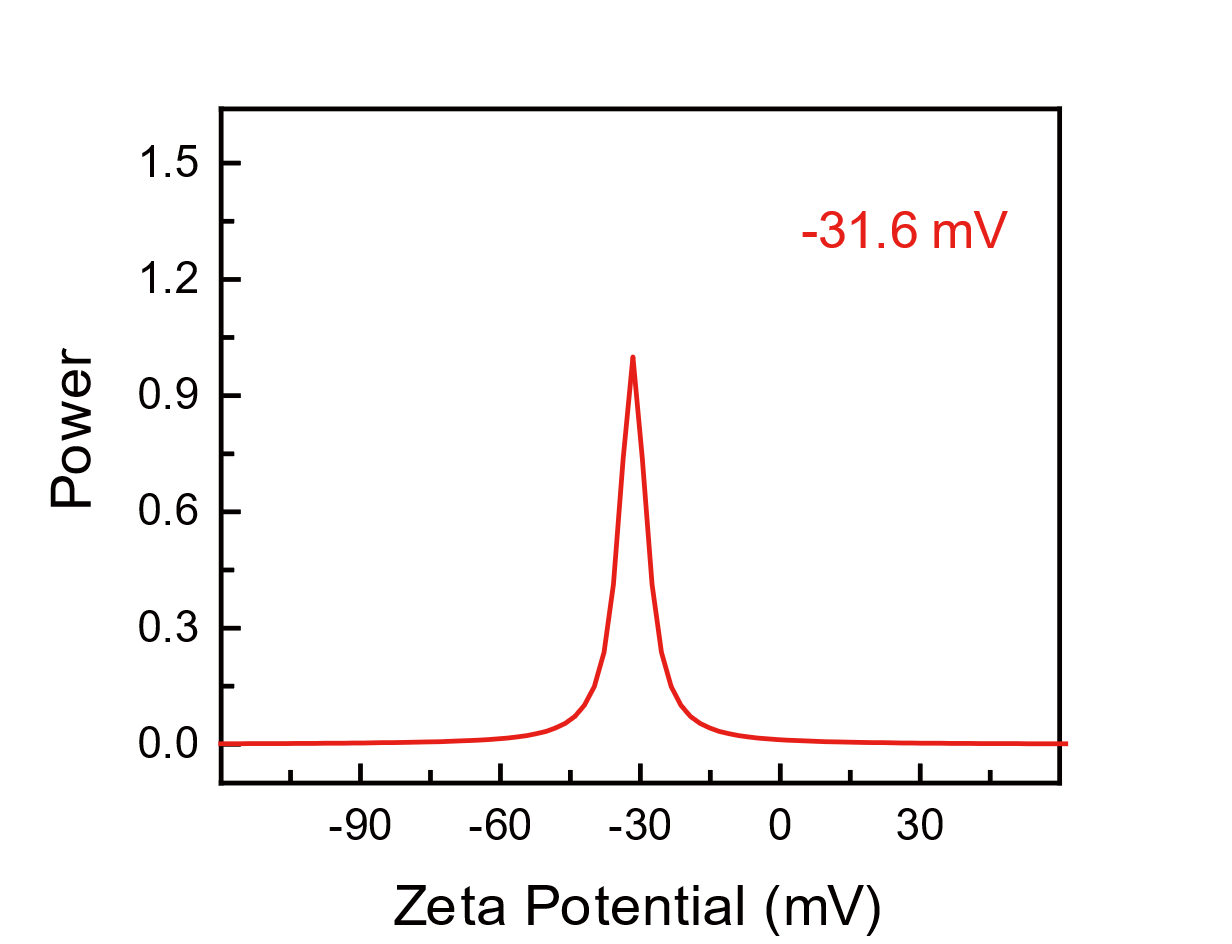


**Fig. S7.** Zeta potential of Gd-HPMA12.


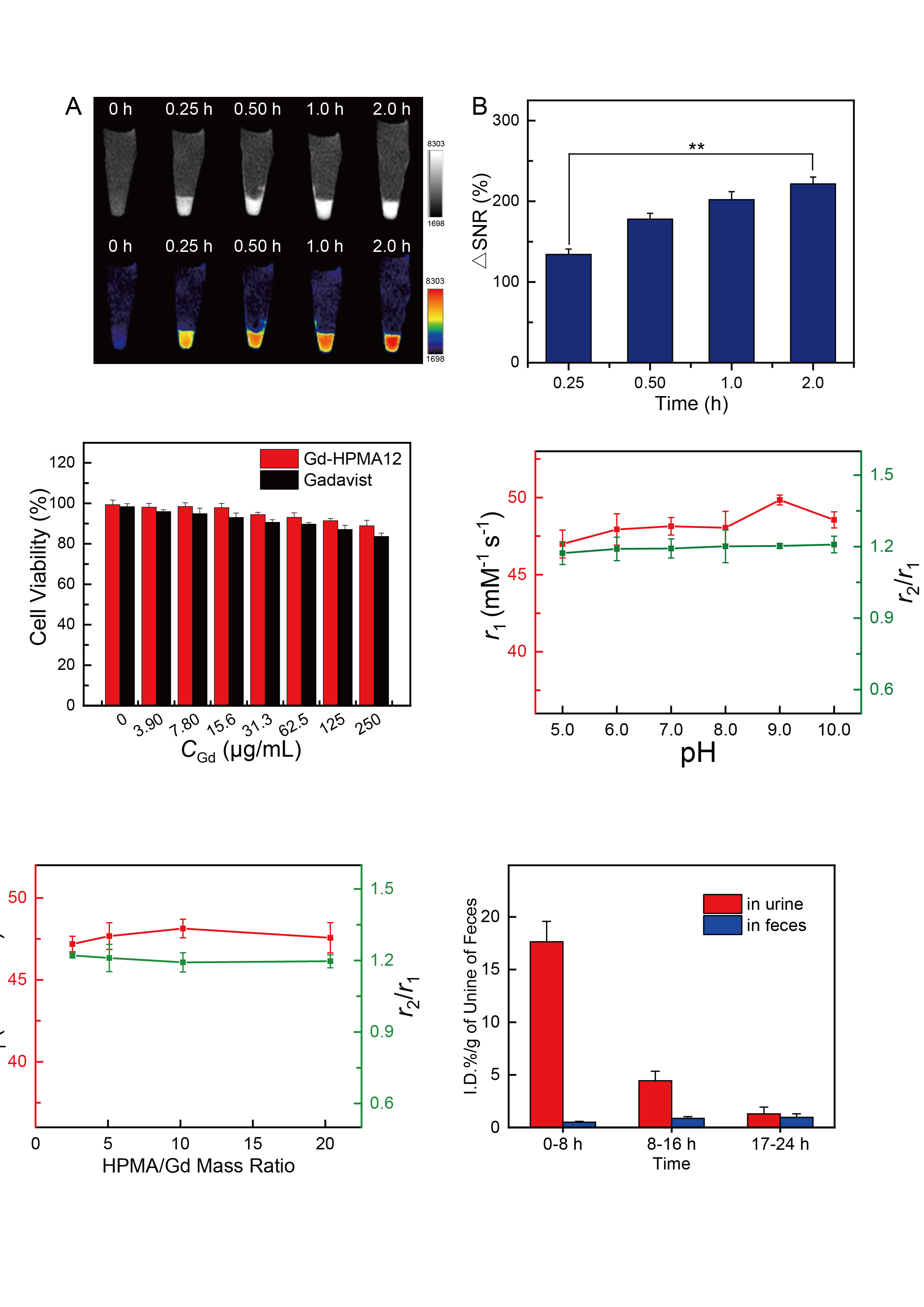


**Fig. S8. MRI of cancer cells *in vitro*.** (**A**): *T*_1_-weighted MR images of 4T1 cell pellets (slice orient: sagittal) incubated with Gd-HPMA12 at various incubation time (3.0 T). (**B**): ΔSNR of the MR images in A. **P < 0.01. Mean ± SD, *n* = 3.


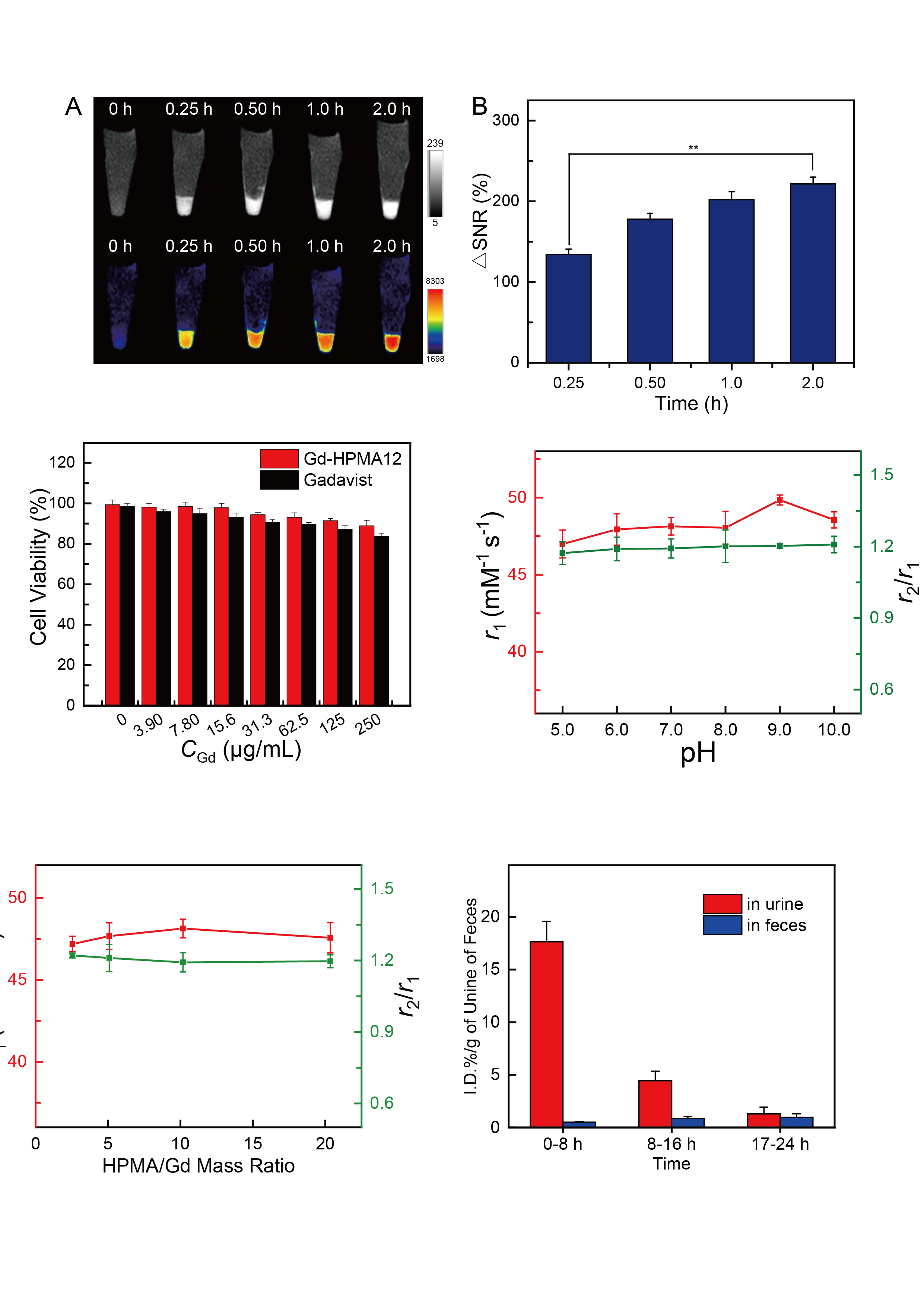


**Fig. S****9.** Viabilities of 4T1 cells treated with Gd-HPMA12 compared with Gadavist® in a Gd concentration range of 0-250 µg/mL. Mean ± SD, *n* = 3.


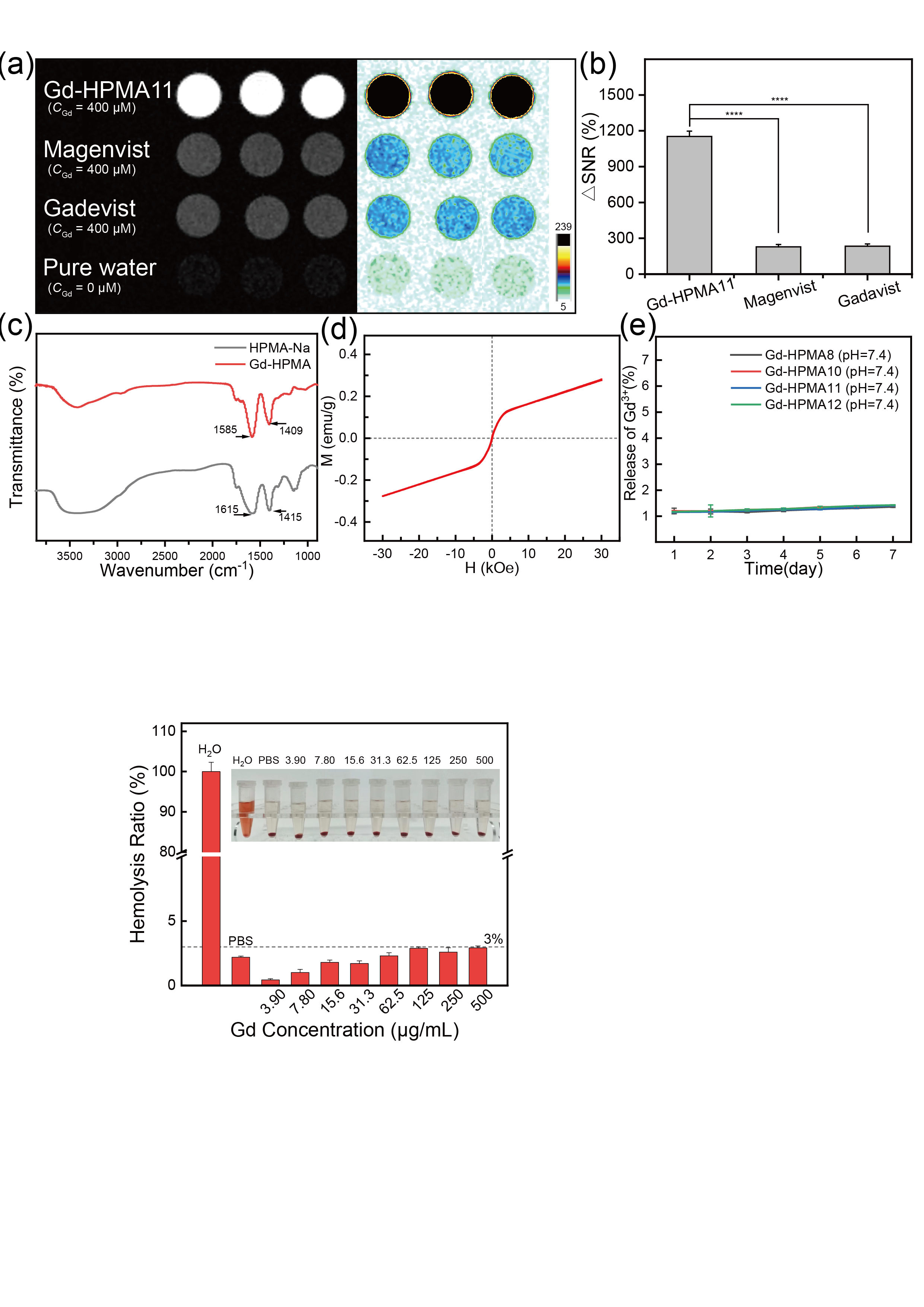


**Fig. S10.** Hemolysis ratio induced by Gd-HPMA12 in a Gd concentration range of 0-500 µg/mL compared with pure water and PBS. Mean ± SD, n = 3.


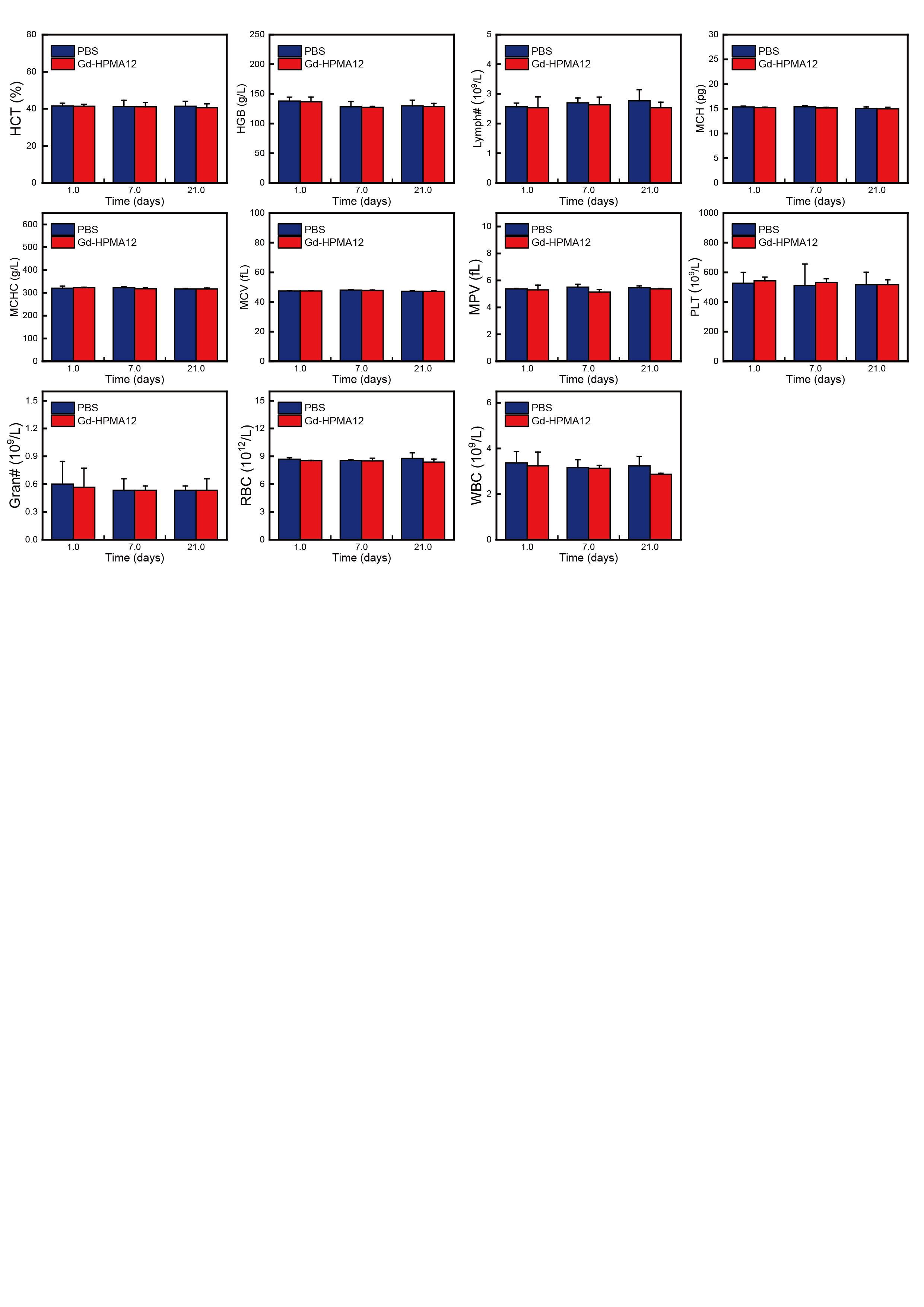


**Fig. S11.** Blood routine analyses of heathy mice at day 1.0, 7.0, or 21 post-injection (*i.v.*) of PBS, or Gd-HPMA12 (Gd dosage = 5.0 mg/kg). Mean ± SD, n = 3. The blood routine analyses include the following indicators: hematocrit (HCT), hemoglobin (HGB), lymphocyte count (Lymph#), mean corpusular hemoglobin (MCH), mean corpusular hemoglobin concerntration (MCHC), mean corpusular volume (MCV), mean platelet volume (MPV), platelet count (PLT), neutrophil ratio (Gran#), red blood cell (RBC), and white blood cell (WBC).


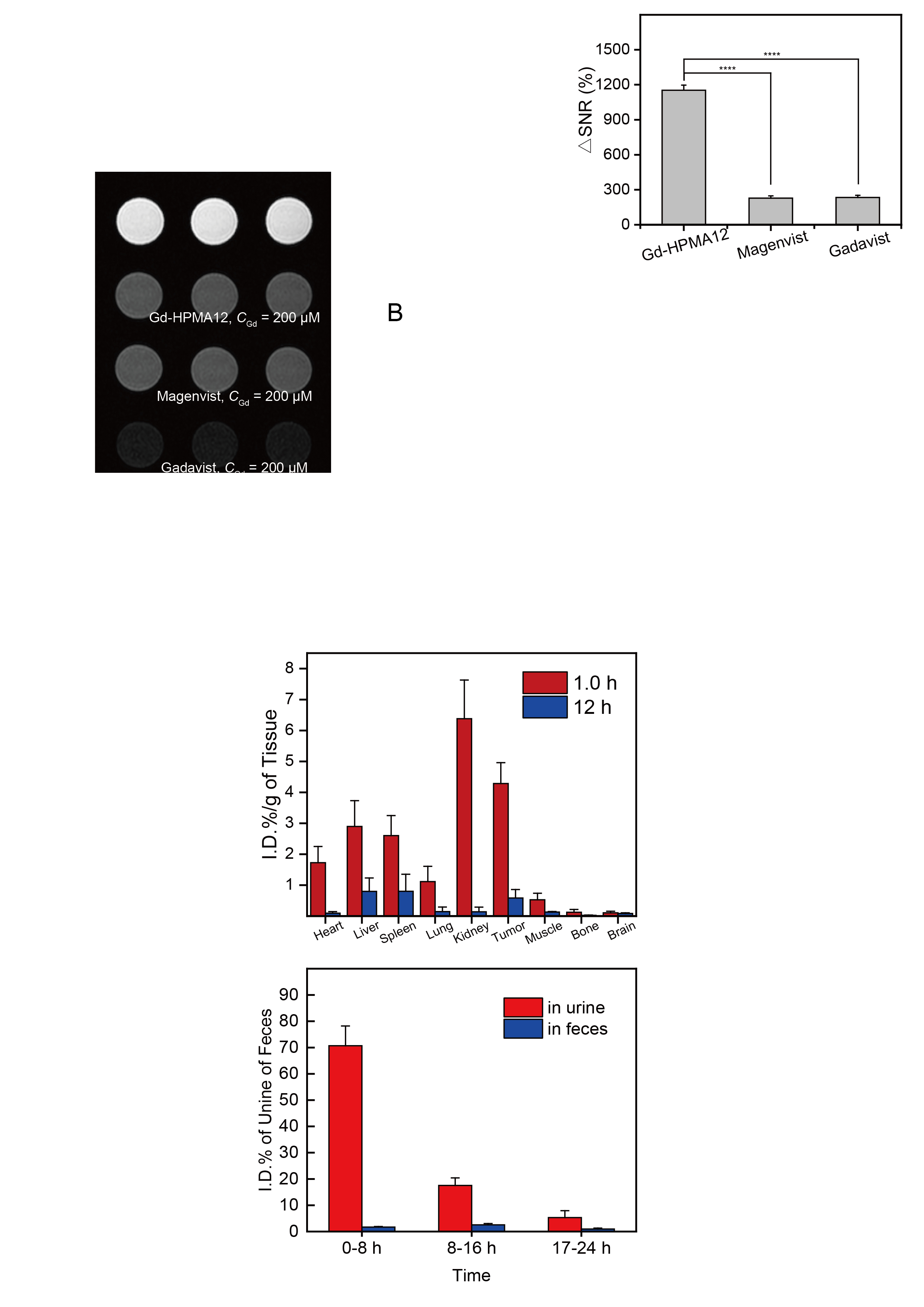


**Fig. S12.** Excreted Gd content in urine or feces of healthy SD mice within 24 h after *i.v.* injection of Gd-HPMA12. Gd dosage = 5.0 mg/kg. Mean ± SD, *n* = 3.


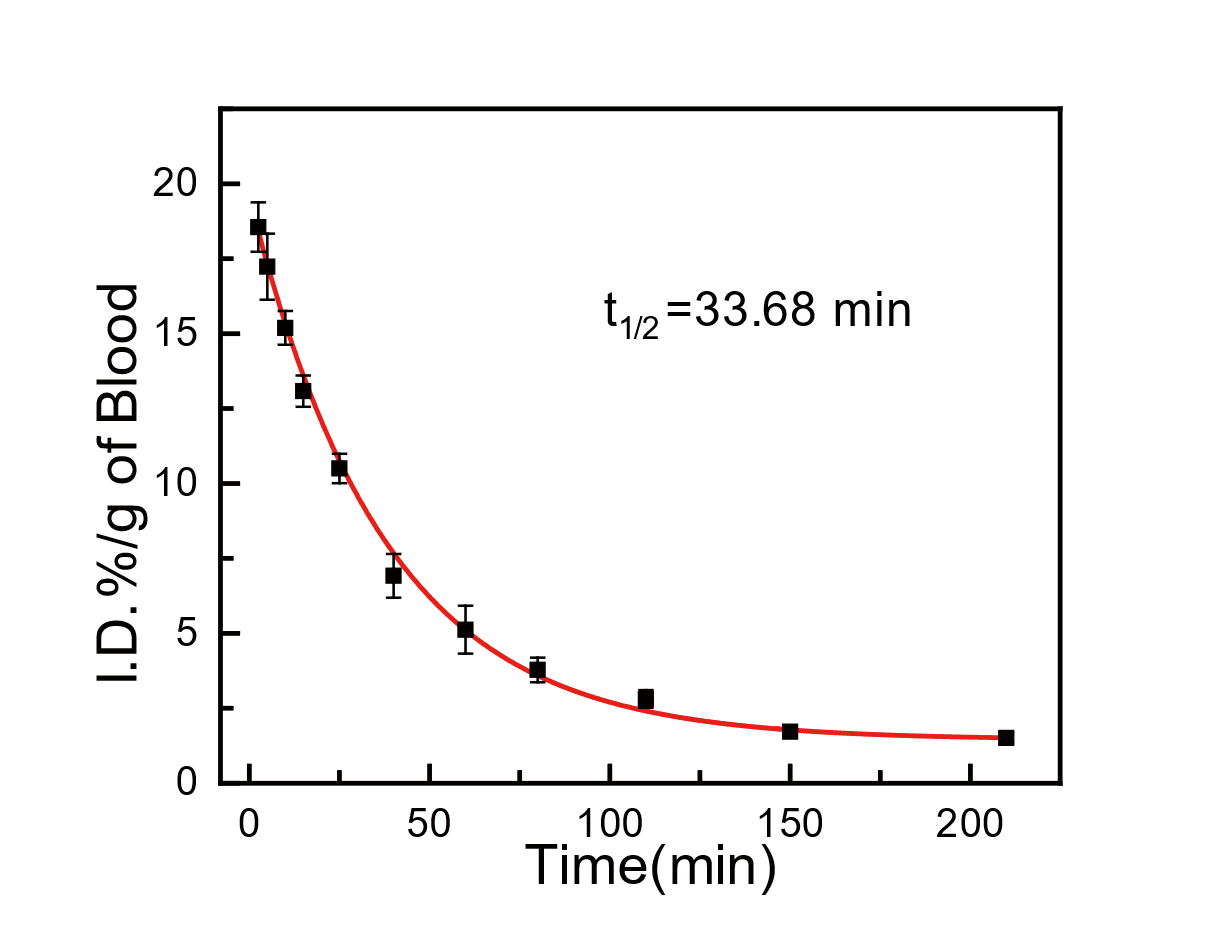


**Fig. S13.** Blood clearance profiles of Gd-HPMA12 in healthy Balb/c mice by tracking the Gd concentration in blood at different time intervals after *i.v.* injection (*n* = 3). Gd dosage = 5.0 mg/kg. Mean ± SD, *n* = 3.


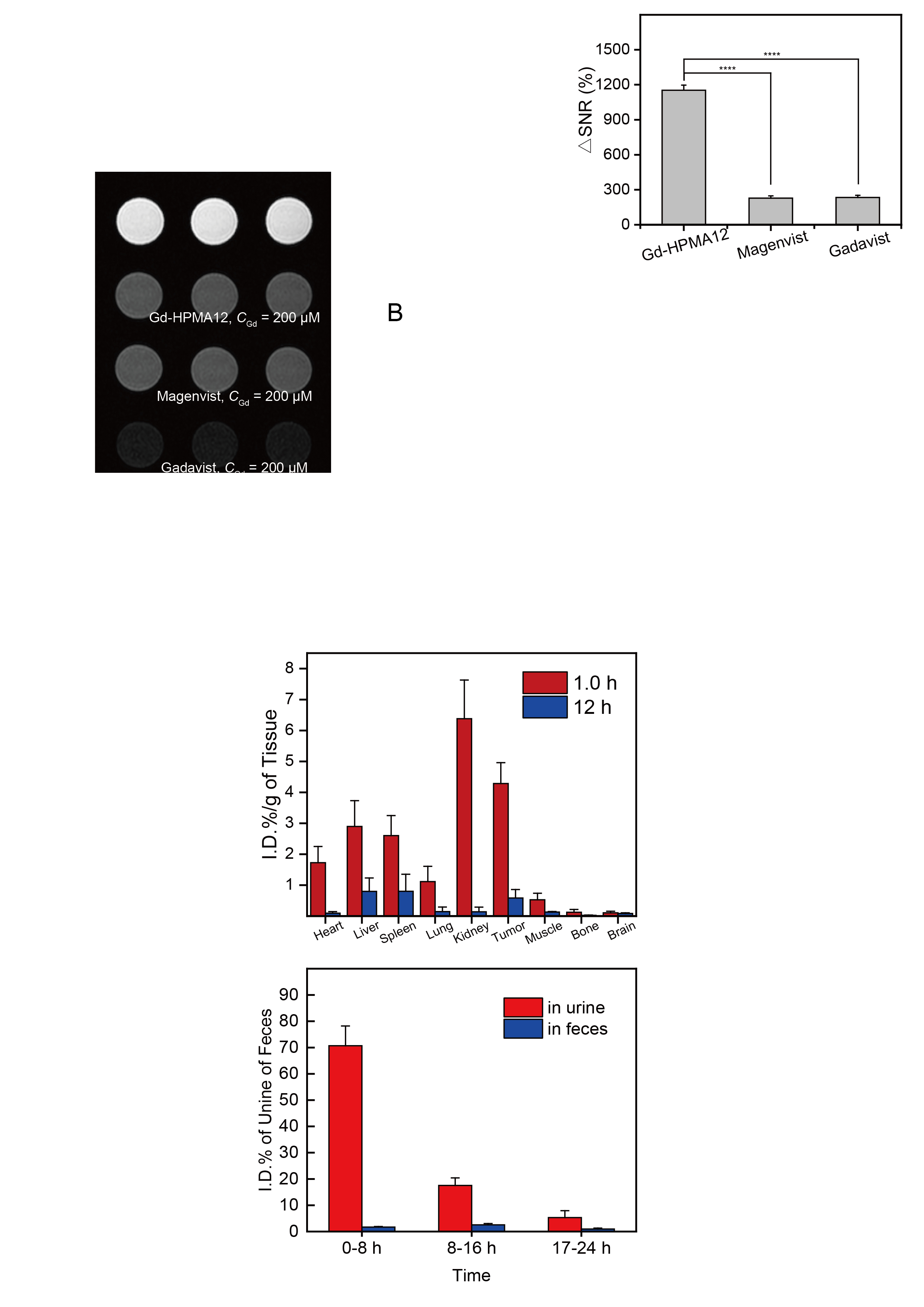


**Fig. S14.** Biodistribution of Gd level in 4T1 tumor-bearing mice at 1.0 or 12 h post-injection of Gd-HPMA12 *via* tail vein. Gd dosage = 5.0 mg/kg. Mean ± SD, *n* = 3.


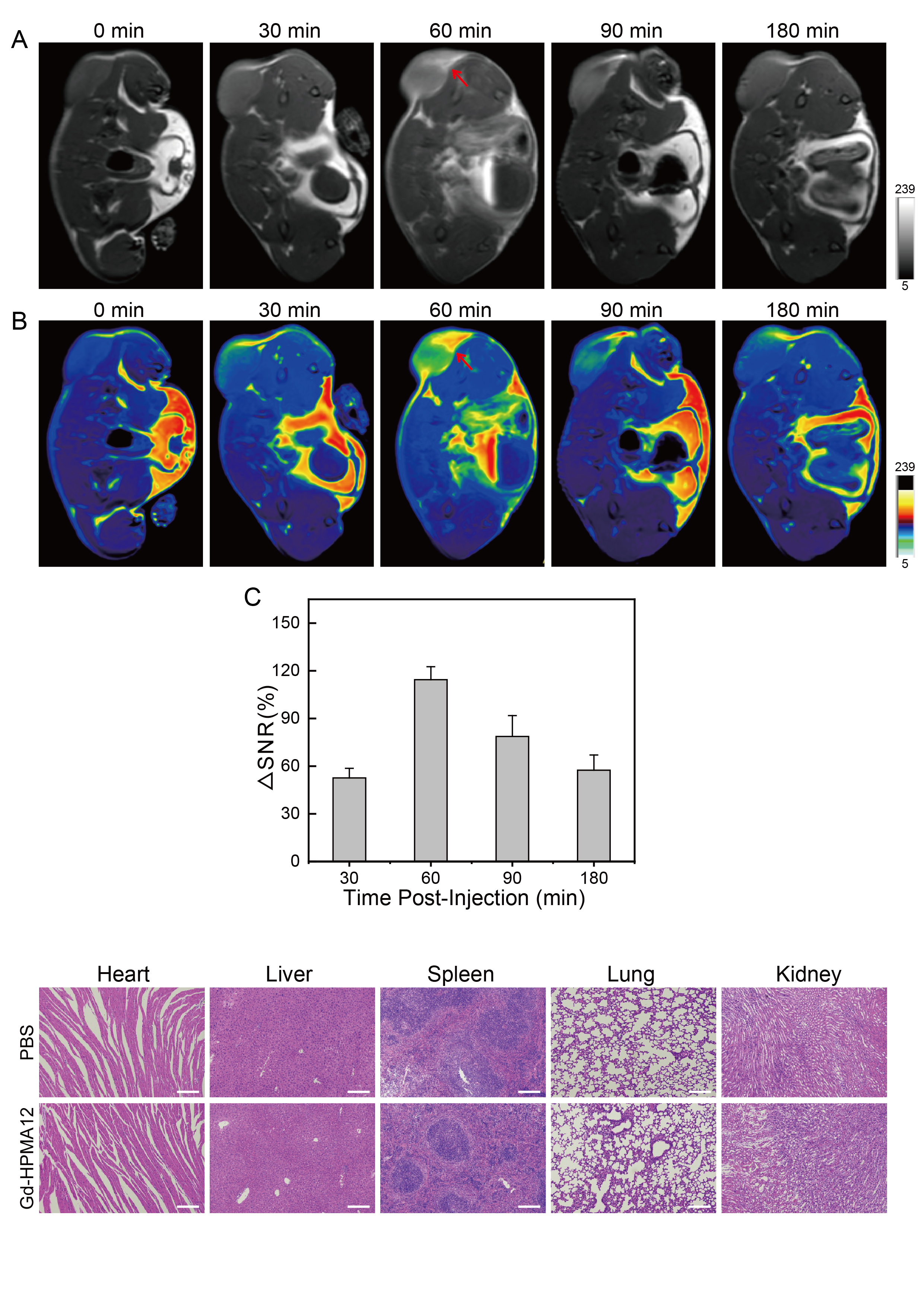


**Fig. S15.** Histological analyses of main organs (H&E staining) obtained from healthy mice at day 2.0 post-injection (*i.v.*) of PBS, or Gd-HPMA12. Gd dosage = 10.0 mg/kg. Scale bar = 100 µm.


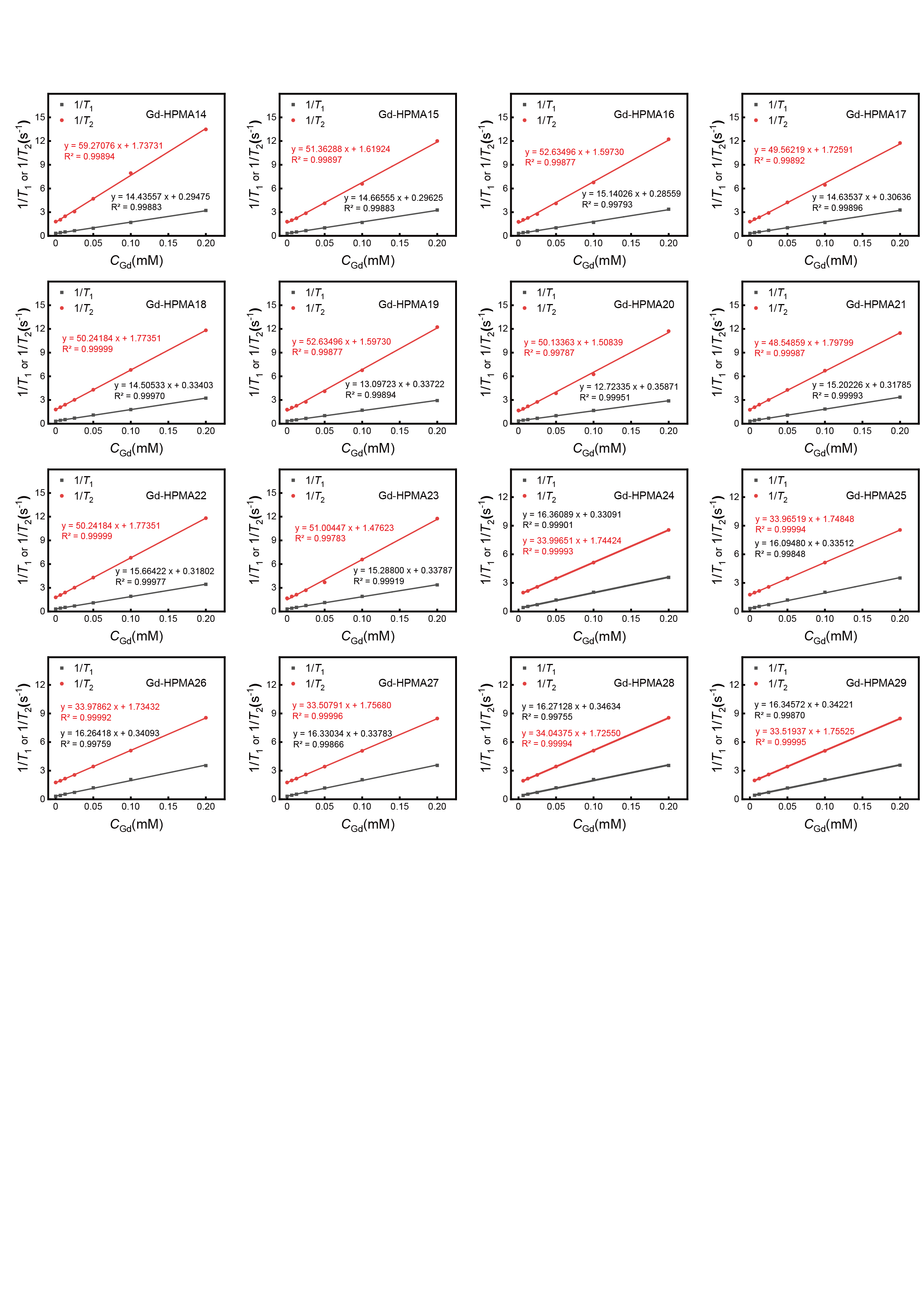


**Fig. S16.** 1**/***T*_1_ or 1/*T*_2_ relaxation rate plotted as a function of *C*_Gd_ for Gd-HPMA14-29 at 7.0 T.


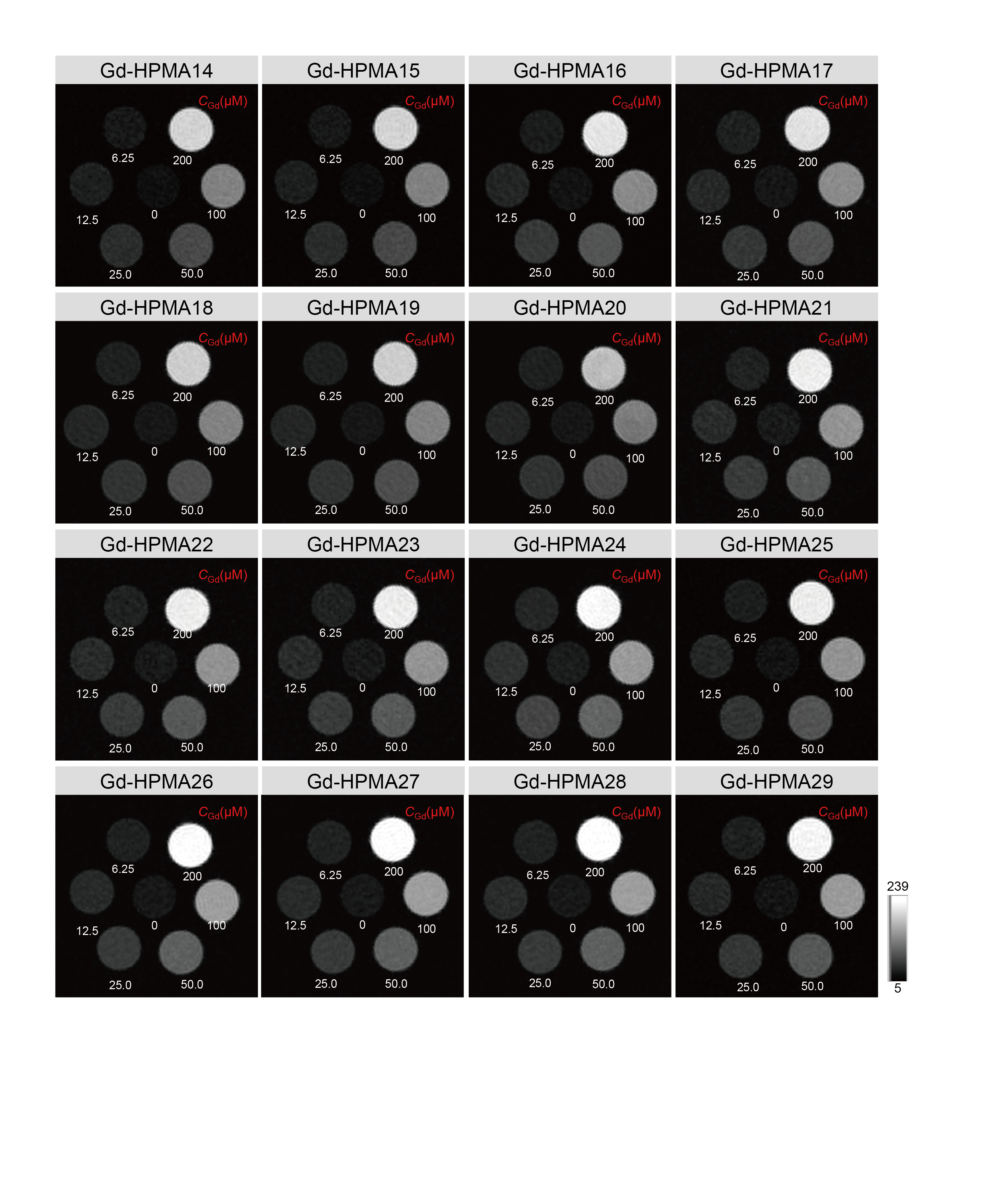


**Fig. S17**. The black & white images of *T*_1_-weighted MR images of Gd-HPMA14-29 with various *C*_Gd_ (0 ~ 200 μM) observed by a 7.0 T clinical MRI system.


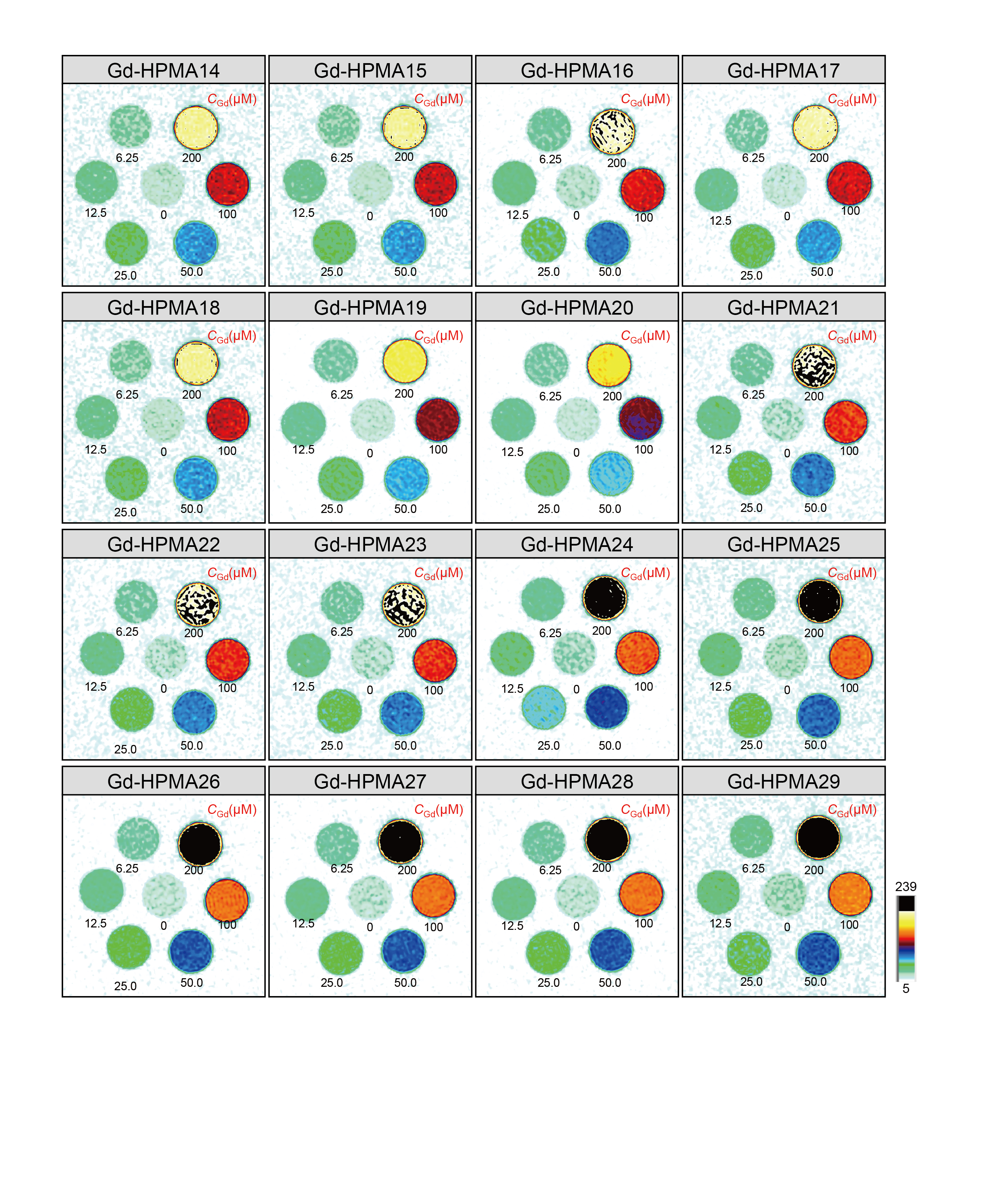


**Fig. S18**. The pseudo-color images of *T*_1_-weighted MR images for Gd-HPMA14-29 with various *C*_Gd_ (0 ~ 200 μM) observed by a 7.0 T MRI scanner.


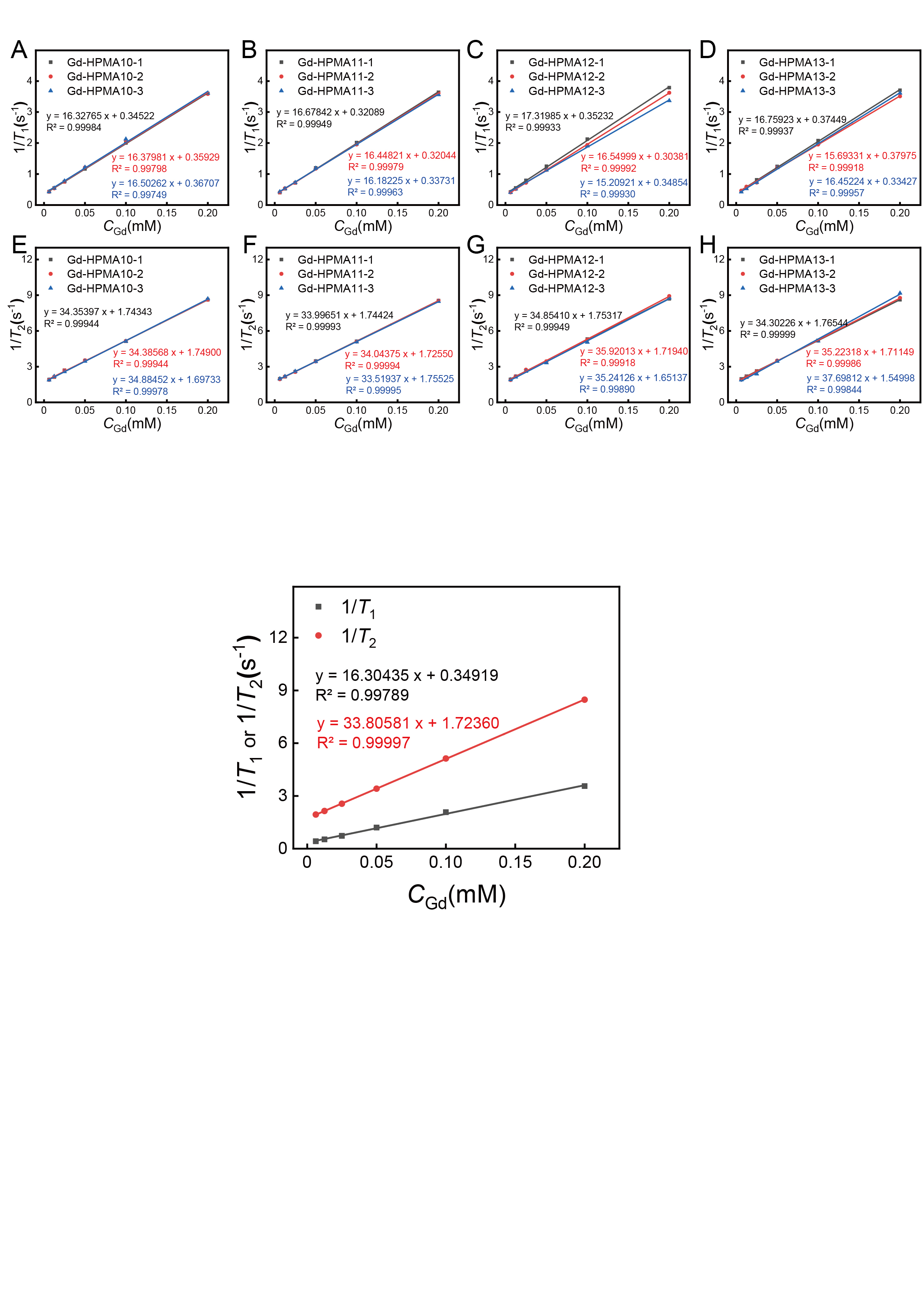


**Fig. S19.** 1***/****T*_1_ or 1/*T*_2_ relaxation rate plotted as a function of *C*_Gd_ for Gd-HPMA30. Magnetic field = 7.0 T.


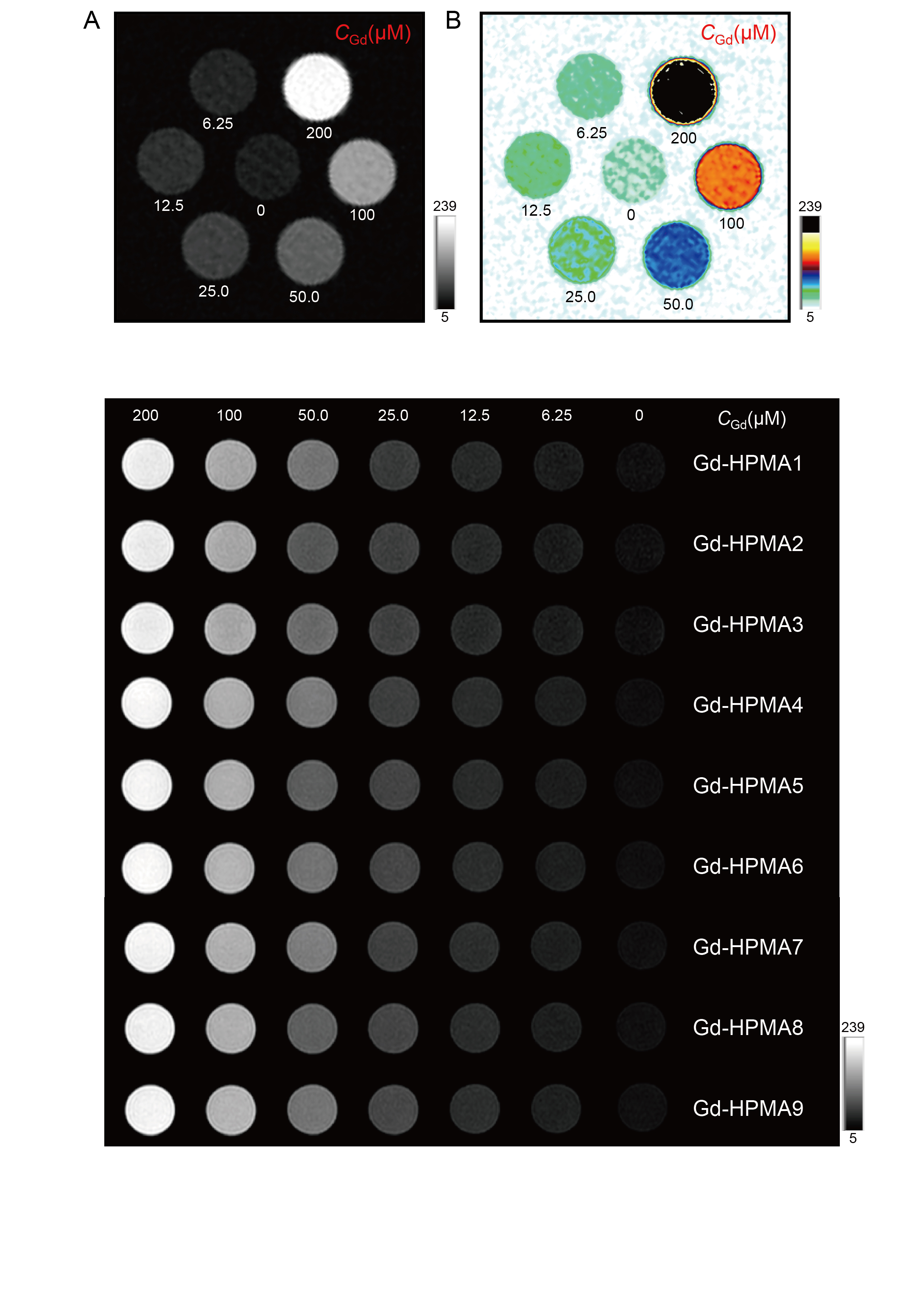


**Fig. S20**. The black & white and corresponding pseudo-color images of *T*_1_-weighted MR images for Gd-HPMA30 macrochelate with various *C*_Gd_ (0 ~ 200 μM) observed by a 7.0 T MRI scanner.
